# Population genomics, demography, and circum-Baltic connectivity of Early Medieval southwestern Finland

**DOI:** 10.1186/s13059-026-04277-7

**Published:** 2026-09-15

**Authors:** Ronan James O’Sullivan, Ulla Nordfors, Nelli-Johanna Saari, Kerttu Majander, Jussi-Pekka Taavitsainen, Kati Salo, Adam B. Rohrlach, Elina Salmela, Thiseas C. Lamnidis, Luca Traverso, Johannes Krause, Sanni Peltola, Päivi Onkamo

**Affiliations:** 1 Department of Biology, University of Turku, Turku, Finland; 2 Department of Archaeogenetics, Max Planck Institute for Evolutionary Anthropology, Leipzig, Germany; 3 Department of Archaeology, University of Turku, Turku, Finland; 4 Globe Institute, University of Copenhagen, Copenhagen, Denmark; 5 Department of Archaeology, University of York, York, UK; 6 Department of Environmental Sciences, University of Basel, Basel, Switzerland; 7 Department of Archaeology, University of Helsinki, Helsinki, Finland; 8 School of Biological Sciences, University of Adelaide, Adelaide, Australia

**Keywords:** Early Medieval Finland, Bioarchaeology, Ancient DNA, Genetic shift, Finland, Sweden, Gotland, Identical-by-descent, IBD, Biobank

## Abstract

**Background:**

Knowledge of Early Medieval Finland (1050–1250 CE) relies primarily on archaeological evidence, as contemporary sources are scarce. The available evidence indicates two distinct cultural-economic zones: coastal and inland. Using newly generated genomic data from 34 ancient individuals alongside modern Finnish genomes, we characterise Late Iron Age and Early Medieval ancestry in southwestern Finland, reconstruct demographic patterns, and place individuals within a circum-Baltic relatedness network.

**Results:**

Early Medieval ancestry in inland southwestern Finland was very similar to that of present-day inhabitants. Ancient coastal and inland individuals were genetically indistinguishable, whereas modern coastal populations showed substantially more Scandinavian ancestry, and less Baltic ancestry compared to their ancient counterparts. IBD (identity-by-descent) analyses also indicate a major genetic shift in the coastal zone since the Early Medieval Period. Effective population size increased throughout the study period and was ~ 13,000 by 1250 CE. IBD links between Scandinavia and Early Medieval Finland align with known archaeological connections. Furthermore, we identify IBD links between individuals from Early Medieval Finland and victims of the Kronan warship sinking.

**Conclusions:**

We demonstrate nearly a millenium of population continuity in the inland zone of southwestern Finland, contrasted by a large, contemporaneous genetic shift in the coastal zone. This ancestry shift corresponds with documented medieval emigration from Sweden to Finland. The regional population rapidly expanded during this time period, likely due to new agricultural practices and favourable climatic conditions. Our circum-Baltic IBD network indicates that southwestern Finland was firmly embedded into the wider, pre-modern Baltic world.

**Supplementary Information:**

The online version contains supplementary material available at https://doi.org/10.1186/s13059-026-04277-7.

## Background

Bioarchaeological studies of pre-modern northern Europe, especially those which utilise ancient DNA (aDNA), are dominated by discussions of the Neolithic, Bronze, Roman Iron, and Viking Ages [1–7]. These studies collectively comprise thousands of ancient individuals’ genomes. In comparison, only 26 ancient genomes with genome-wide data of sufficient coverage for analysis (≥ 30,000 on-target SNPs) have been published from Finland [8–14]. However, an additional 103 ancient mitochondrial genomes have also been published [13]. This lack of autosomal genomes is partly due to the rapid decomposition of unburnt human remains caused by the region’s acidic soil [14]. Despite this paucity, the genomes that have been published suggest a complex population genetic history for pre-modern Finland. Studies demonstrate that Sámi-like ancestry (the Sámi are the indigenous people of Fennoscandia) once extended much further south than where it is found today [8, 9] a conclusion further supported by linguistic, archaeological, and historic evidence [15–18]. Only three other studies of ancient autosomal genomes from Finland have been published [10–12]: these focus on long-term population continuity between Medieval and present-day Pirkanmaa, Finland [10], the genetics of a potential decapitation victim [11], and a bioarchaeological reconstruction of the life history of a 16th-century Sámi individual [12]. However, a common finding across most of these studies is individual-level mobility between pre-modern Scandinavia and Finland.

This dearth of understanding of pre-modern Finnish genomics stands in stark contrast to the extensive literature on the population genomics of modern Finland [19–23]. The modern research is a direct result of multiple large health studies of different Finnish population cohorts, the data for which are now stored at the biobank of the Finnish Institute for Health and Welfare (in Finnish: *Terveyden ja hyvinvoinnin laitos*, henceforth, THL—[24]). As a consequence, the present-day population genetic structure of Finland is very well understood. One of the most striking results from these population-level studies is the existence of a sharp genetic division between southwestern and northeastern Finns [19, 22, 25, 26], with *F*_*st*_ values similar to what is more often observed between geographically separate populations, such as Britain and Germany [19, 21]. This genetic division likely arose due to permanent, wide-spread agriculture becoming established far earlier in southwestern Finland, ~ 4000 years ago, compared to eastern Finland, ~ 500 years ago [27, 28]. The southwestern-northeastern genetic divergence was likely compounded by successive genetic bottlenecks and drift in the east [19, 20]. Additionally, consistent emigration from Sweden to Finland since at least the 1200s CE has led to Scandinavian admixture in western Finland as well as a sizable Swedish-speaking, coastal resident minority (*Finlandssvenskar* [29]) who are themselves admixed with, but genetically distinct from, Finnish-speaking Finns [25]. As a consequence, Finlandssvenskar display a stronger genetic affinity to the Swedish population than the rest of the Finnish population [30].

Pre-modern Finland was not an isolated region. In the Early Iron Age (c. 500 BCE–400 CE), influences from the Baltic region and Scandinavia are evident in artefact types and burial customs [31]. Linguistic and archaeological evidence suggest that during this period, groups of Proto-Finnic speakers migrated from the area of present-day Estonia to the coastal regions of Finland [32]. Over time, these groups mixed with the existing inhabitants of the region who were likely Proto-Sámi speaking, contributing to the emergence of the population that would centuries later be identified as “Finns”. During the Merovingian Period (c. 550–800 CE), eastern connections became more visible, particularly through the appearance of Permian bronze ornaments originating from the western Ural region [33]. Archaeological evidence from the period 500–700 CE also demonstrates that trans-Baltic connections between Finland and Scandinavia became common. Prestige items such as Germanic swords, found in high-status male burials in Finland, have been interpreted as diplomatic gifts or tokens of allegiance from Scandinavian chieftains to their Finnic allies [31, 34]. These alliances may have been reinforced through marriage ties or fosterage [35, 36]. Such alliances helped integrate leading families from coastal Finland into broader Baltic networks of power and exchange. These relationships continued into the Late Iron/Viking Age (c. 800–1050 CE), when ties between Finland and Scandinavia became increasingly visible in the archaeological record. Finnish-made or Finnish-influenced goods, such as ceramics and dress ornaments, have been found at the trading site of Birka in Sweden, indicating interaction over the period [37]. Imported goods such as glass beads, Islamic, Anglo-Saxon, and Germanic coins, as well as Baltic and Scandinavian jewellery and weapons, indicate active Finnish participation in trans-Baltic and Eurasian exchange systems, with furs likely serving as a primary trade commodity [31, 38, 39]. Indeed, the Finnish word for money, *raha* also means “fur pelt” [40]. The connections were likely facilitated not only by mutual economic interests, but also by ongoing political and familial relationships throughout the northern Baltic region. Isotope analyses provide clear evidence for sustained interactions and mobility between southwestern Finland and Sweden during the Viking Age and Early Medieval period, as individuals buried in Finland show isotopic signatures indicating childhood origins in Sweden and vice versa [41, 42].

Throughout the first millennium CE, the southwest was the core settled area of Finland due to its favourable climate and strategic location along Baltic maritime routes which connected Northern Europe with more distant regions. From roughly 1050–1250 CE onwards, connections across the Baltic Sea evolved into more structured forms of interaction as the emerging Kingdom of Sweden expanded eastward into what is today southwestern Finland. This era, which marks the Early Medieval period in Finland, witnessed the beginning of political and ecclesiastical integration of southwestern Finland into the western European cultural sphere and the Roman Catholic Church [31, 39, 43, 44]. While archaeology has uncovered aspects of material culture from this time period [35, 43–47], the surviving written accounts of the inhabitants of southwestern Finland, and of Finland as a whole, are often fragmentary and either shaped by external viewpoints (i.e. Novgorod Chronicles, Hagiography of Saint Erik), or composed much later (Agricola’s New Testament from 1548). Furthermore, no primary, natively-written accounts of life in Early Medieval Finland exist today.

Traditionally, this time period has been known as the Finnish “Crusade Period”, reflecting the long-held assumption that the Swedes conducted violent military campaigns to forcibly convert local populations to Christianity. However, this is a misnomer as the three so-called crusades are mostly regarded by modern scholars as later medieval literary constructions rather than verifiable historical events. The “crusades”, while being papally-sanctioned, most likely involved small-scale military activities aimed at consolidating Swedish rule over eastern Fennoscandia, rather than large-scale campaigns of conquest [48–50]. Due to these issues, scholars have proposed more historically and archaeologically grounded alternatives, such as the Late Viking Age to emphasize the continuity of cultural practices from the Iron Age [45] or the Early Medieval Period [43, 44] to reflect the gradual emergence of Christian institutions, political structures, and external influences without presupposing military conquest. In light of this, we refer to this era here as the Early Medieval Period.

**Fig. 1:**
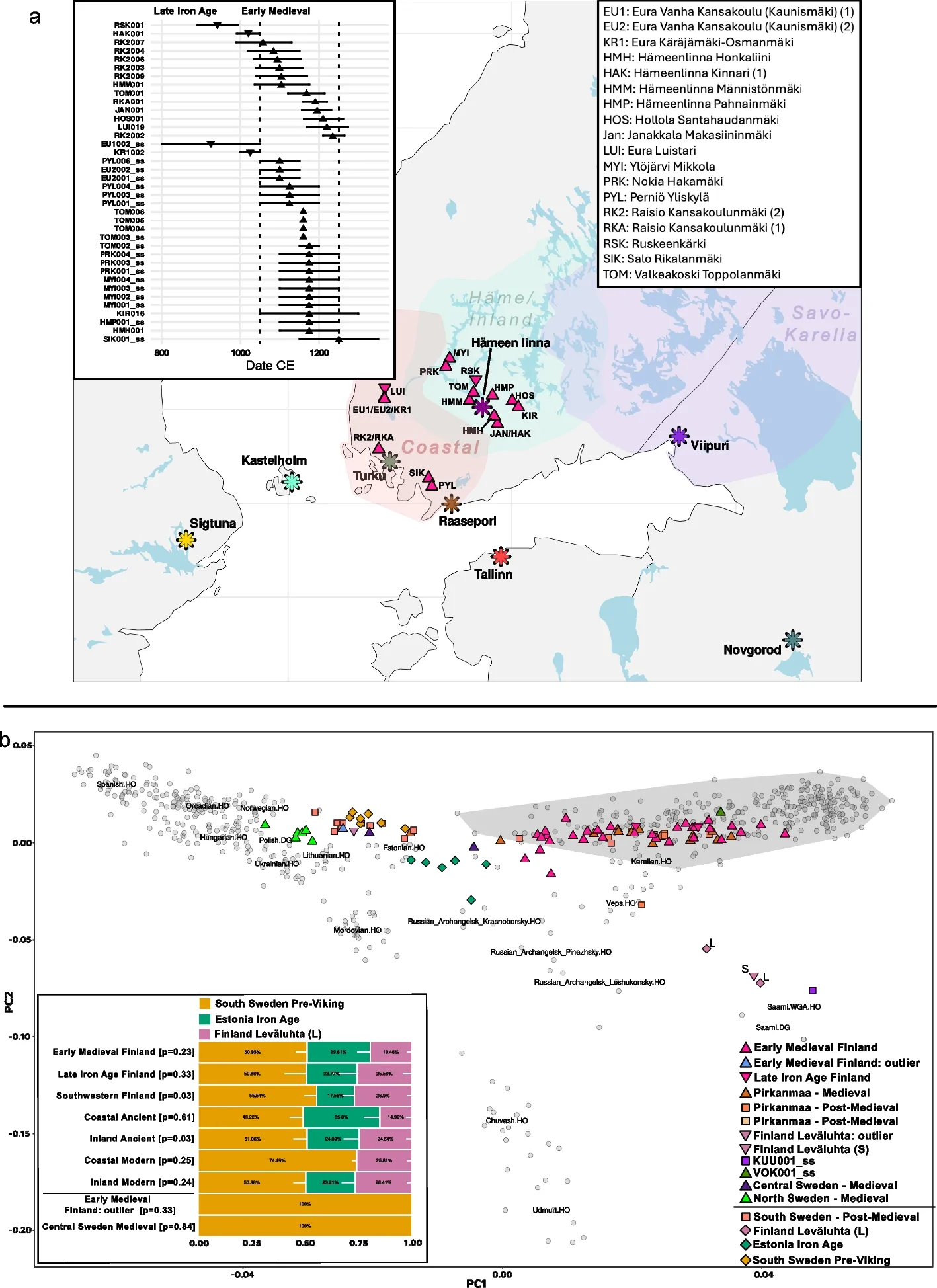
**a** Map of the three major settlement zones in southern Finland circa 1000 CE. Major trade centres (Sigtuna, Tallinn, Viipuri) and castles (Kastelholm, Turku, Raasepori, Hämeen linna, Viipuri, Novgorod) are indicated with bold text and asterices. Upright and down pointing pink triangles indicate the Early Medieval and Late Iron Age archaeological sites, respectively, where the 38 individuals in this study were found. From left to right, inset plots are: (inset top-left) point estimate and one standard error (if available) for both ^14^C-dated and contextually-dated individuals. As in (**a**), triangle orientation indicates historical period; (inset top-right) Key for archaeological site abbreviations; **b** PCA of 27 present-day Eurasian populations from west of the Ural Mountains (grey) with all 26 previously published ancient individuals with genome-wide data from Finland projected, as well as the 34 non-contaminated genomes analysed in this study. Present-day Finnish genetic variation (grey-shaded polygon) represented by a convex hull generated from 276 whole-genome sequences of modern Finnish Biobank donors. The proximal ancestry sources for individuals from Late Iron Age and Early Medieval Finland (determined using qpAdm) are also projected (diamonds). The “L” or “S” next to light pink polygons indicate if that Leväluhta individual was from Lamnidis et al. [8] (L) or from Sikora et al. [9] (S). The “_unc.” suffix indicates an uncertain dating for that group. Note, three individuals (HMM001, RK2003, LUI019) labelled as “Early Medieval Finland” fall outside of the convex hull of present-day genetic variation. However, f-statistics confirmed their affinity with Finnic populations. Hence, they are not treated as outliers. Merged genotypes are filtered on their shared Human Origins positions; Inset bottom-left: Proximal ancestry components of main analysis groups determined using qpAdm. The presented models are the simplest, passing, non-anachronistic, most plausible models. Error bars represent one standard error. We present models where *p* ≥ 0.01. Right populations were Ethiopia_4500BP.DG, Russia_Krasnoyarsk_BA.SG, EEHG (Eastern European Hunter-Gatherer), WEHG (Western European Hunter-Gatherer), Steppe, and EN_LBK. See Additional file 1: Tables S5, S6 for full model output

Finland was not a unified political entity during the Early Medieval period, but instead consisted of several culturally and economically distinct regions (Fig. 1a). Archaeological sources suggest at least three major settlement zones in the southern part of present-day Finland: the southwestern coastal zone, consisting of the present-day administrative regions of Varsinais-Suomi and Satakunta, which had exhibited the strongest material ties to Scandinavia already during the Viking Age; the eastern Savo-Karelian sphere; and the inland zone of Häme marked by a hybrid cultural profile with influences from both western and eastern traditions [31, 46] (Fig. 1a). Based on archaeological evidence, it has been suggested that the Häme Finns expanded eastward into the Savo and Karelian regions during the seventh and ninth centuries [51, 52], and mixed with local forager communities, possibly related to present-day Sámi, that already inhabited the areas [52]. During this period, the mixed population in the Karelian Isthmus gradually formed a distinct group known as the Karelians, shaped in part by increasing influence from the Novgorodian sphere [51, 52].

In this study, we generated genome-wide target capture data from 38 ancient individuals found at 17 archaeological sites (17 and 21 individuals from the coastal and inland zones of southwestern Finland, respectively, Additional file 1: Table S1) spanning a 450-year time period (800–1250 CE). We analysed 34 of these individuals alongside both ancient and present-day published data in order; (1) to characterise the genetic ancestry of Late Iron Age (LIA: 800–1050 CE) and Early Medieval Period (EM: 1050–1250 CE) southwestern Finland, relative to other ancient and present-day groups; (2) to determine if the ancient cultural-economic divide between the coastal and inland zones was also reflected in the zones’ respective genetic compositions, and; (3) to provide complimentary genomic evidence for archaeologically-attested mobility between southwestern Finland and other regions around the Baltic Sea. We demonstrate that despite some cultural and economic differences between the coastal and inland zones, they formed a genetic clade. Ancestry modeling and IBD-sharing patterns with modern-day Finns indicate genetic population continuity within the inland zone since at least the Late Iron Age. However, the same analyses suggest substantial genetic discontinuity in the coastal zone post-1300 CE, most likely driven by increased immigration to Finland from Sweden. Haplotype-based estimates of effective population size, *N*_*e*_, for the region indicate a rapid increase in population size: around 800 CE, the midpoint estimate of *N*_*e*_* was* ~ 2500 (95% confidence interval [CI]: 2203, 3007) while by 1250 CE, it was ~ 13,000 (95% CI: 7247, 19,484). This increase is in accordance with archaeological evidence for higher agricultural output driving a demographic expansion. Finally, we contextualize individuals within a circum-Baltic IBD network in order to understand the place of what is today Finland within the wider political, economic, and cultural landscape of pre-modern northern Europe.

## Results

### Evidence for inland persistence and coastal change in southwestern Finland

Using PCA, we visualized the genomes of the LIA/EM individuals with respect to both present-day Eurasian (Fig. 1b) and Finnish (Additional file 2: Fig. S1) genetic variation. Nearly all individuals lie within or close to present-day Finnish variation (Fig. 1b), and most lie within that observed in southwestern Finland (Additional file 2: Fig. S1). The Pirkanmaa Medieval and Post-Medieval individuals (from the inland region) published in [10] displayed a near-complete overlap in PC space with the LIA/EM individuals (Fig. 1b). Similar to the Pirkanmaa groups, f-statistics showed that LIA/EM displayed the highest genetic affinity with Karelians, Veps, and Finns from the Human Origins panel (Additional file 2: Fig. S2; Additional file 1: Table S2; their Table S3), but neither group was cladal with these modern populations, nor with present-day southwestern, eastern, or northern Finns (Additional file 2: Fig. S3, Additional file 1: Table S3). Similarly, when grouped by geography rather than time, the Coastal_ancient and Inland_ancient groups formed a clade to the exclusion of 12 present-day and 18 ancient groups (Additional file 2: Fig. S4, Additional file 1:Table S4). Proximal modelling of the ancient ancestry of both zones also points towards them being the same population (Fig. 1b—inset, Additional file 1: Tables S5, S6). This confirms that the economic and cultural differences ascribed to the residents of the LIA/EM coastal and inland zones does not manifest as a genetic difference. Although not formally distinguishable, Inland_ancient displayed positive f4 statistics with a source of Siberian ancestry, more similar to more eastern Uralic-speaking populations (e.g., Udmurts) and Sámi, while Coastal_ancient were more attracted to Scandinavians (Additional file 2: Fig. S5; Additional file 1: Table S7). This trend in Siberian ancestry likely reflects the southwest-northeast genetic division that has been observed in the present-day Finnish population [21]. However, properly studying this genetic division among individuals from Finland’s ancient DNA record will not be possible until genomes of ancient individuals from eastern Finland are published.

**Fig. 2:**
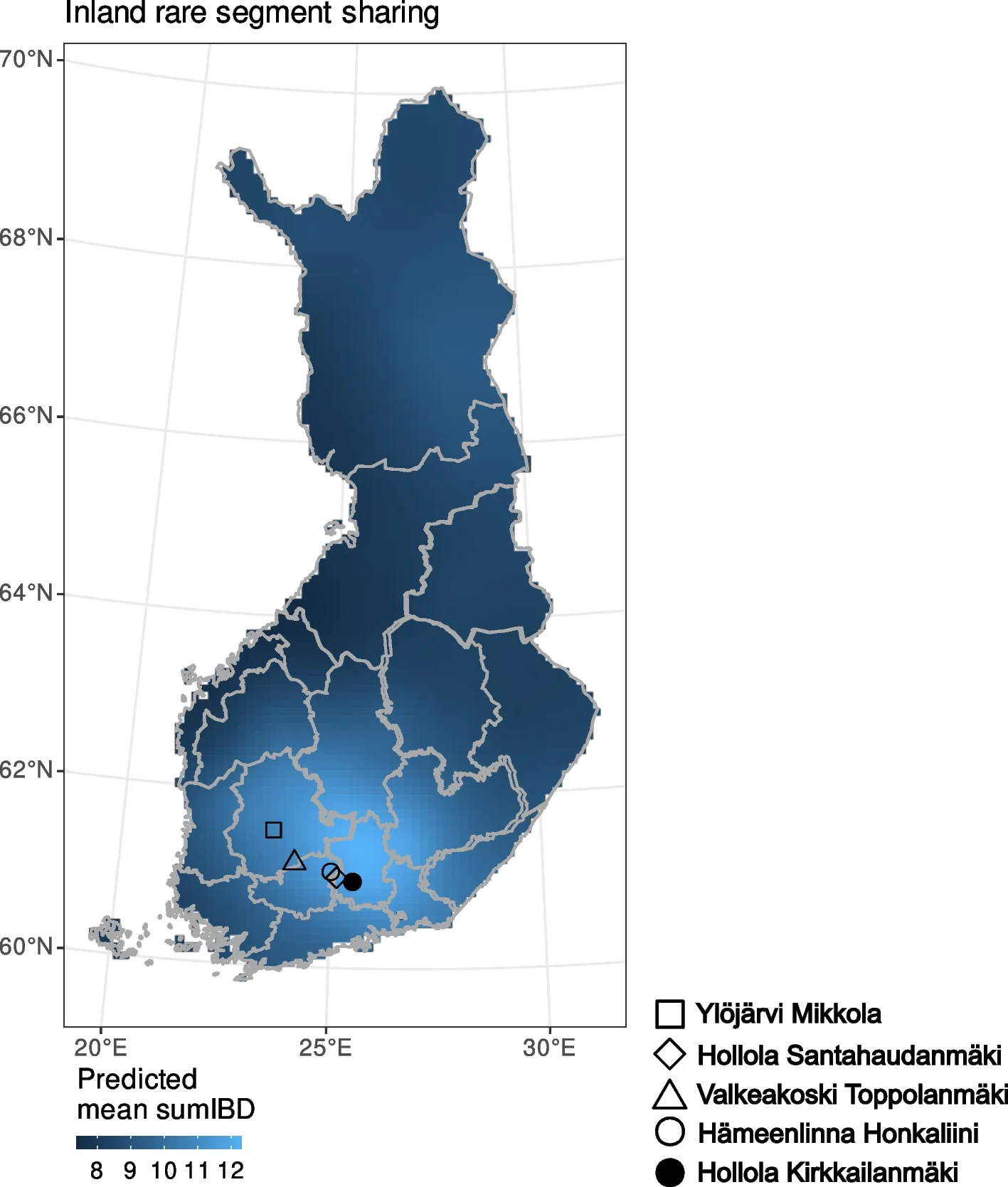
Interpolated IBD connectedness between (a) ancient Inland zone individuals and present-day Finnish THL Biobank donors. Normalized sums of IBD segment lengths between ancient-modern pairs are calculated at the level of the municipality separately for the Inland and Coastal zones. The municipality average of this figure is used for kriging. Lighter shading corresponds to higher IBD connectedness, and darker shading corresponds to lower IBD connectedness. Common segments refer to IBD segments that an ancient individual shares with more than one modern individual. Segments that an ancient individual shares with only one modern individual are here referred to as rare segments. Modern Finnish administrative region sample sizes are in Additional file 1: Table S14

How past and present communities are related to each other, either culturally or genetically, is the implicit focus of much bioarchaeological research. Nordfors et al. [10] have previously shown ~ 800 years of population continuity within the inland zone. The three sites studied in [10] are located along a 30 km stretch of the same glacial ridge. We used patterns of rare IBD segment sharing between ancient and modern residents of the inland zone to push this estimate back to nearly ~ 1000 years ago (Fig. 2—lighter shading corresponds to higher IBD connectedness, darker shading corresponds to lower IBD connectedness). Crucially, we used a more geographically dispersed set of independent ancient genomes, thus demonstrating that this pattern of IBD sharing is a regional phenomenon occurring across a much larger area. Taken altogether, these results recapitulate those of genetic continuity across a remarkably long time period presented in [10]. As such, the continuity is not an anomaly restricted to a single set of uniquely linked burial sites but rather a feature of the regional gene pool. The reduced mobility implied by our results is reflected in migration rates for the region derived from present-day genomic data [20].

Strikingly, this same pattern of genetic continuity is not observed in the coastal zone. While the ancient coastal population was cladal with the ancient inland population, proximal qpAdm modelling suggested a genetic shift in the coastal population since the EM period. Coastal_modern yielded a 24.97%-unit increase in Scandinavian-derived ancestry compared to the ancient coastal group. There was also a reduction in observed Baltic-derived ancestry relative to all other Finnish groups, either ancient or modern (Fig. 1b—inset bottom-left). Additional models, designed to test if Coastal_modern could be modelled using just Coastal_ancient as a source, confirmed that the Coastal_modern population displayed an increased affinity towards a source of Scandinavian ancestry relative to the Coastal_ancient population (Additional file 1: Table S8). In stark contrast to the inland zone, there was no discernible spatial pattern of rare or common IBD sharing between individuals from the ancient coastal zone and present-day Finns from the same region (Additional file 2: Fig. S6a, b). Together, both allele- and haplotype-based approaches indicate a genetic shift in the coastal zone of southwestern Finland between the end of the Early Medieval Period (~ 1250 CE) and the modern day.

### Circum-Baltic connections and local demography

**Fig. 3:**
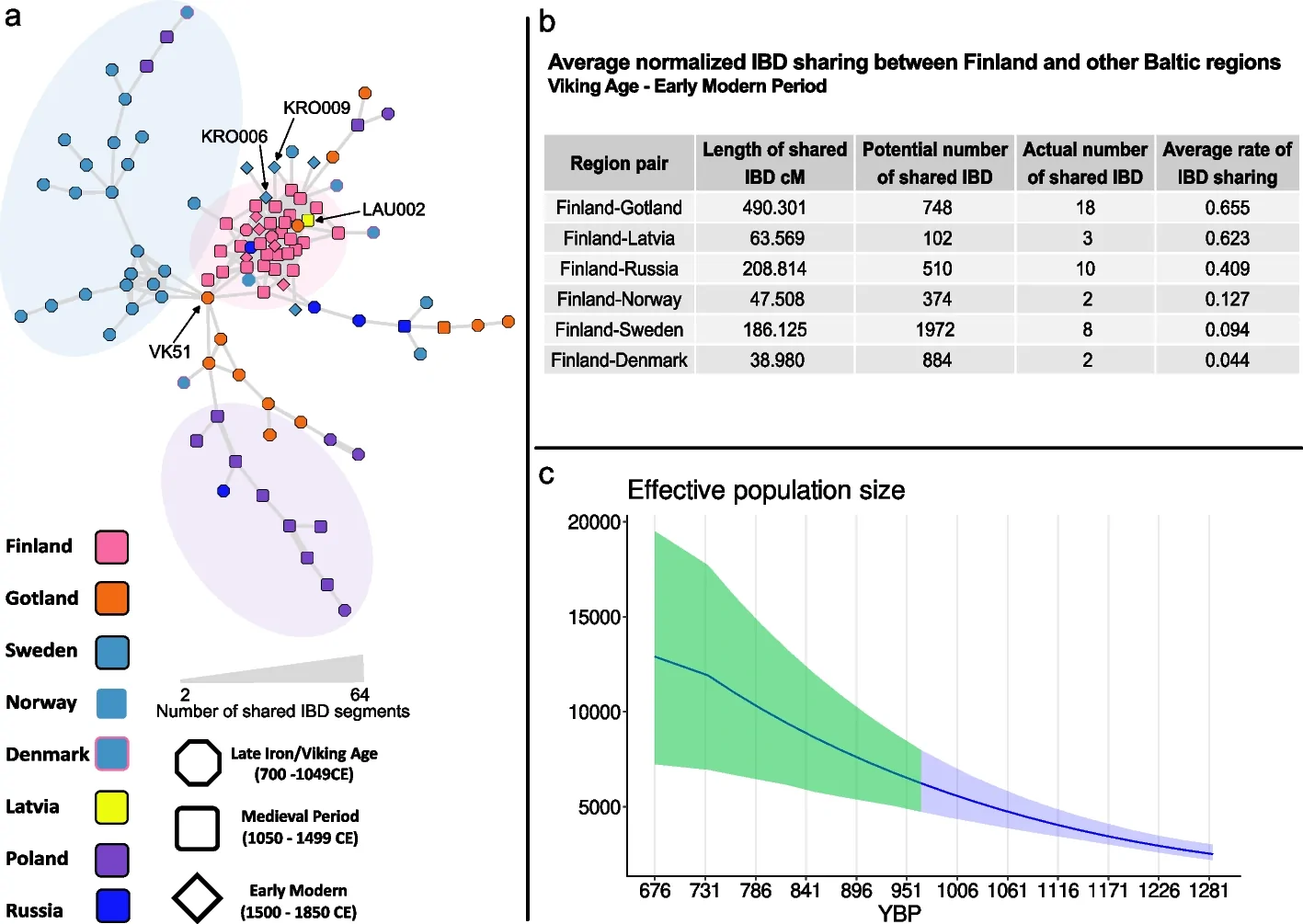
Haplotype-based demographic results: **a** A contiguous circum-Baltic Identity-By-Descent (IBD) network showing inter-regional connectivity between ancient individuals ranging from the Viking Age to the Early Modern Period. Segments of shared IBD ≥ 8 cM (centiMorgans) called using ancIBD and visualised using Cytoscape. Edge widths (grey lines) are scaled by the total number of IBD segments shared by a pair of individuals, with a thicker edge indicating that the pair share a greater number of IBD segments than those pairs with thinner connecting edges. If two individuals are not connected, then they do not share IBD segments. A prefuse force directed layout is applied to the entire network graph (contiguous and satellite networks—Additional file 2: Fig. S8) which causes individuals that share more IBD segments to be pushed more closely together. Individuals colour-coded by their region of burial and age indicated by shape of node; **b** Average IBD sharing between Finland and one of six circum-Baltic regions. IBD sharing is normalized by the number of potential pairs of individuals between regions that could share IBD segments, thus, accounting for sample size; **c** The effective population size (N_e_) of Late Iron Age and Early Medieval Finland inferred from patterns of shared IBD segments called using TTN_e_. The central blue line is the midpoint estimate Ne, bounded by upper and lower 95% confidence intervals. The green portion of the ribbon represents estimates inferred from the observed IBD segments shared between individuals while the blue portion represents predicted values based on the observed pattern but with no underlying data. YBP stands for Years Before Present

Using *ancIBD*, we recovered a contiguous IBD network of 266 shared segments between 103 ancient individuals from regions with Baltic Sea coastlines that dated from the Viking Age until the Early Modern Period. The maximum number of IBD segments ≥ 8 cM (centiMorgans) shared between two individuals was 64, with the maximum sum of IBD length between two individuals being 3336.92 cM (Fig. 3a, Additional file 1: Table S9). This network consisted of three main groups; Scandinavian (blue), Slavic (purple), and Finnish (pink). An ancient individual from Gotland connected the ancient Finnish group with the other two groups. When controlling for the potential number of connections between regions, Finland shared the most IBD connections with Gotland, Latvia, and Russia (Fig. 3b, Additional file 1: Table S10), and, to a lesser extent, with Norway, Sweden, and Denmark.

*TTN*_*e*_ (*Time-Transect N*_*e*_ [53]) estimated an effective population size (*N*_*e*_) during the LIA/EM that ranged from ~ 2500 (95% CI: 2203, 3007) at the start of the LIA to ~ 13,000 (95% CI: 7247, 19,484) by the end of the EM Period (Fig. 3c, Additional file 1: Table S11). While we believe the younger *N*_*e*_ estimates to be relatively robust (see Discussion), the older LIA estimates must be interpreted with caution since they are based on fewer individuals and, thus, fewer coalescence events. Consequently, these earlier estimates are more likely to be affected by *TTN*_*e*_’s regularization algorithm. We examined runs of homozygosity (ROH) and found that while many individuals displayed small sums of ROH, none displayed long stretches (Additional file 2: Fig. S7, Additional file 1: Table S12). This indicates some level of distant parental relatedness but not of consanguinity between close relatives—a pattern consistent with the small population sizes we estimate.

## Discussion

In this study, we analysed the genomes of 32 individuals from the Early Medieval Period and 2 from the Late Iron Age of southwestern Finland. We demonstrated that despite the economic and cultural differences between southwestern Finland’s inland and coastal zones, the Early Medieval populations in these regions were genetically similar, but have since experienced different trajectories. The inland zone demonstrates nearly a millennium of genetic continuity between medieval times and present day. This result expands the phenomenon of long-term genetic persistence reported in [10] from the local to regional scale. In contrast, the coastal zone has experienced a genetic shift of the population during the same period, with present-day ancestry of the region’s inhabitants being enriched for Scandinavian-derived ancestry relative to other ancient and modern Finnish groups. These results provide further nuance to discussions concerning the formation of the present-day Finnish gene pool.

### Continuity and change at the regional scale

The long-term stability of the inland zone’s gene pool while the coastal zone experienced major Scandinavian influx is striking, particularly so given that the two zones are separated by less than ~ 150 km (Fig. 1a). Based on previous genetic work [29], the most likely source of this Scandinavian ancestry was almost certainly Swedish immigrants from Svealand and Norrland to coastal areas of Finland. Given that we observe similar ancestry profiles for both the coastal and inland zones during the LIA/EM (Fig. 1b—inset), the large-scale immigration events that caused the discontinuity must mostly post-date the thirteenth century. However, it is possible that some of the migration predates the thirteenth century, as suggested by isotope studies [42]. If the immigrants did not mix with the local population, or if individuals with such ancestry were not represented in the samples, no corresponding genomic signal would be observable. Historical research, drawing on evidence such as place names and the medieval division of villages into parishes according to Finnish and Swedish law, has proposed that Swedish migration occurred mainly in the late 13th and early 14th centuries CE [29, 31, 54, 55]. The political integration of Finland into the Kingdom of Sweden began in the 1200s and continued throughout the 1300 s/1400 s CE as Sweden contested for control of Finnish territory with the Republic of Novgorod and, later, the Grand Duchy of Moscow. Integration involved the establishment of fortifications throughout southwestern Finland: the castles of Kastelholm, Turku, Hämeen linna, and Raasepori were all founded during this period.

The genetic shift/discontinuity we describe implies that formal political integration was accompanied not only by fortification, but also by emigration from medieval Sweden to its new eastern province (Finnish territories were known as “*Österland*”). However, the genetic continuity observed in the inland zone between Early Medieval times and the present day indicates that immigration was concentrated along the southwestern coast, and did not extend very far into the hinterland. There may be several non-mutually exclusive reasons for this dichotomy. For example, Swedish immigrants may have preferentially settled near centres of familiar secular and ecclesiastical power (the administrative and ecumenical centers were located in coastal southwestern Finland). Alternatively, maintaining economic, political, and familial ties with Sweden may have been easier for coastal settlers due to proximity to maritime communication and trade routes. The genomic data indicate limited admixture between coastal Swedish-speaking groups and inland Finnish groups, despite documented coexistence and interaction [55, 56]. This pattern suggests that the consolidation of Swedish authority in inland regions proceeded primarily through administrative and sociopolitical structures, rather than through substantial population movement inland. Coupled with the low inland zone immigration rate inferred from present-day Finnish genomic data [20], this could provide both an explanation for how the present-day genetic difference between the inland and coastal zones came to be, as well as how it was maintained. Such a dynamic between geographically proximate groups is not without precedent: two Merovingian Period communities from the Vienna Basin maintained a genetic barrier for roughly 200 years despite their shared Avar culture [57].

A limitation of our study is that it can only place a lower bound on the date when large-scale immigration from Sweden likely took place. Since all the burial sites of the ancient individuals whose genomes we analyzed mostly form two geographic groups (Fig. 1a) and all date from before large-scale immigration (Additional file 1: Table S1), we cannot examine phenomena such as clinal variation between the coastal and inland zones, nor pulses of migration. In order to investigate these questions, future work should focus on individuals buried at locations along the SW-NE axis between the two zones, and dated to the fourteenth-sixteenth centuries. While the current population of Finlandssvenskar (Swedish-speaking Finns) is almost certainly descended from those who migrated during the fourteenth-sixteenth centuries, a somewhat later chronology has sometimes been suggested [31, 54, 55]. Time-depth modelling of haplotypes shared between present-day Swedes and Finlandssvenskar is required to definitively confirm the timeline.

### Population demographics

Using a transect of individuals who shared IBD segments from across our study period, we reconstructed a time series of estimates of the effective population size (*N*_*e*_). *N*_*e*_ appeared to increase over time from 2511 (95% CI: 2203, 3007) at 1281 YBP to 12,900 (95% CI: 7247, 19,484) at 676 YBP. The earlier date corresponds to about 50 years (750 CE) before the oldest calibrated ^*14*^*C*-date of the oldest individual in this study (EU1002_ss) and the later date to about 50 years (1274 CE) after the lower age estimate of the youngest (LUI019). The trajectory of increase and *N*_*e*_ estimates are in line with previous estimates derived from genomic data for present-day Finns [20]. (20) showed that by ~ 1250 CE, the *N*_*e*_ of the inland zone was ~ 12,000. The difference in estimates is negligible as the estimate of [20] is within the 95% confidence interval of ours. Any true difference is likely due to our analysis using ancient genomes which, being temporally closer to the study period, have experienced fewer recombination events than present-day genomes. Thus, temporally distant signals of IBD segment sharing (and, by extension, coalescence events and *N*_*e*_) can be detected in ancient data that would not be observed in estimates derived from present-day genomes.

The increasing trajectory of *N*_*e*_ coincides with the introduction of new plough technologies to Finland, most likely from Scandinavia and the Baltics, during the LIA/EM [58]. The new ploughs allowed for agricultural development of the heavy clay soils that cover much of southwestern Finland [58]. This followed an already-occurring increase in agricultural output for southwestern Finland that began during the first millennium CE [59]. Radiocarbon modelling suggests an increase in population size starting around 700 CE [27]. Additionally, macrofossil and pollen evidence indicate a proliferation of open grasslands from the mid-1100s CE [60]. We found no evidence for widespread consanguinity in the LIA/EM population (Additional file 2: Fig. S7, Additional file 1: Table S12) despite the relatively small effective population size. While many individuals displayed short runs of homozygosity, this is likely due to distant parental relatedness which is often observed for groups with small census and effective population size. These results align with previous evidence from the inland zone [10] showing that the Medieval population of the region was outbred, likely with social mechanisms to avoid inbreeding [61].

### Circum-Baltic connections: Finland within a northern European context

Our IBD network demonstrates that the LIA/EM population of southwestern Finland was genetically connected with Viking Age individuals from Sweden, Gotland, Denmark, Russia, Norway, Medieval Latvia, and Early Modern Sweden (Fig. 3a). Individuals fell into three groups that aligned with ancestry and geography: Scandinavian, Slavic, and Finnish. The Finnish group was connected to the other two via a Viking Age individual (VK51) from Gotland (Fig. 3a). A previous study [62] modelled this individual’s ancestry as being 91.7% “Finnish-like”. Three more presumably Gutnish individuals from that study (VK56, VK60, VK475) also displayed large proportions of Finnish-like ancestry, likely being either direct migrants or recent descendants of Finnish migrants. Indeed, Finland displayed higher average normalised IBD sharing with Gotland than with any other Baltic region (Fig. 3b, Additional file 1: Table S10). These genetic connections mirror known archaeological links between Gotland and Finland, with Gotland serving as a major centre of cross-Baltic exchange [63–66]. The genomic and material connections suggest a central role for Gotland in movement and trade between Finland and elsewhere in northern Europe.

Nordfors et al. [10] report shared IBD segments between individuals from medieval Pirkanmaa and Viking Age individuals from Gotland (VK51—the same individual as in this study) and Lofoten. Lamnidis et al. [8] and our study show the presence of genetic outliers (JK1965 and PRK001_ss) with Scandinavian-like ancestry along the south/southwest coast of Iron Age and Early Medieval Finland. Coupled with our circum-Baltic IBD network, these results collectively support archaeologically attested trade, migration, and gene flow between Scandinavia, Finland, and further east into western Russia [35, 37, 66]. The movement of people that is implied by the genetic results, coupled with runic inscriptions found in, or referring to, Finland [67], may provide a tentative historical basis for later literary works that describe Scandinavian expeditions to eastern Fennoscandia, e.g., the Heimskringla saga’s account of King Olav II of Norway raiding the southern Finnish coast.

The high rate of IBD sharing between Finland and Latvia is notable (Fig. 3b), especially so since it is driven by a single person (LAU002, Fig. 3a). LAU002 is a genetically female individual found at the Laukskola burial site along the Daugava river in Latvia, dated to 1031–1147 CE [68]. The community at Laukskola is thought to have spoken a form of Liv, a nearly extinct Finnic language. Grave goods suggest the community was wealthy, likely a result of various trading centres along the river [68]. LAU002’s pattern of IBD sharing with RK2004 and MYI003 suggests a distant relationship, at the closest sixth cousins (Additional file 1: Table S9). Given these three individuals were largely contemporaneous, their distant kinship likely reflects a common ancestor coming from Latvia to settle in southwest Finland, or vice-versa. Connections between Finland and the Baltic region are evident throughout the Iron Age, particularly in women’s clothing, accessories, and jewellery [31, 51], which also suggests that women moved between the regions. The high rate of IBD sharing between individuals from trading towns in Kievan Rus’ (Gnezdovo and Staraya Ladoga) and Finland further underscores the importance of trade networks in pre-modern individual mobility. The retention of the IBD segments in Finland’s Early Medieval population is likely due to a combination of relatively smaller *N*_*e*_, resulting in elevated rates of IBD sharing with respect to other regions. This phenomenon of ‘haplotype retention’ is still observed in Finland today [20]. Additionally, our IBD analysis likely underestimates individual mobility from Scandinavia due to the region’s much larger population size compared to Finland and the eastern Baltic. This result highlights the importance of interpreting IBD networks of ancient individuals not just in light of the prevailing cultural and historical contexts, but also with respect to local population genetic dynamics.

Similarly, we recovered IBD connections between Early Medieval individuals from Finland and two victims of the Kronan warship sinking (KRO006, KRO009) that align with fifth-cousin or greater relationships (Fig. 3a, Additional file 1: Table S9). The Stora Kronan (*The Great Crown* in Swedish) was a flagship of the Swedish navy until it sank at the Battle of Öland in 1675 CE during the Scanian War between Norway-Denmark and the Swedish Empire [69]. A previous study [4] found that KRO006, KRO009, and two more individuals from the Kronan (KRO011, KRO014) displayed increased affinity to a Uralic ancestry source. Our IBD results, coupled with historical Swedish records attesting to officer recruitment for the Kronan from Finland [70], corroborate that these officers either came from Finland, or were descendants of Finnish immigrants to Sweden.

### Siberian and Sámi-related ancestry

All Uralic-speaking populations, except Hungarians, display a significant Siberian ancestral component almost absent from other populations [71, 72]. In Fennoscandia, variation in the proportion of this component is most often interpreted as differential admixture with Sámi-like groups, who carry much higher proportions of Siberian ancestry than their geographic neighbours [4, 10]. It has long been assumed that Sámi-speaking-related groups inhabited areas further south in Finland than today, an interpretation grounded in toponymic evidence [17] and supported by genetic data indicating that some Iron Age individuals display affinities with present-day Sámi populations [8]. We find IBD connections between several individuals from LIA/EM Finland with the same individual with Sámi-like ancestry from Lofoten, Norway (VK518), as do [10]. That study found no temporal shift with respect to affinity with Sámi-like ancestry for the inland region. Given this result, and the temporal and spatial overlap of our study with theirs, we do not repeat their analysis despite our larger sample size. Studying admixture dynamics between the genetically Sámi-related and Finnic-related populations of southwestern Finland would likely require a transect of individuals from the Pre-Roman Iron Age until the beginning of the Merovingian Period.

While our F4 statistics for Siberian affinity show no difference between present-day southwestern Finns and any of our analysis groups, it is interesting to note that the Inland_ancient group displayed greater affinity to Siberian ancestry than the Coastal_ancient group (Additional file 2: Fig. S5). While not significant (all |*z*|-values < 3), the decrease in Siberian ancestry from east to west matches the trend observed in Asian-related ancestry for the present-day Finnish population [19]. This difference becomes stronger when only those sites strictly on the coast (Coastal Strict, see Methods) are analyzed. The establishment of this cline in Siberian ancestry in southwestern Finland likely predates our study period by several centuries and likely demonstrates a long-standing role of the southwestern coast as the link between Finland and the rest of the Baltic region.

## Conclusions

We use ancient DNA from Late Iron Age and Early Medieval individuals to provide a genomic snapshot of pre-modern southwestern Finland and its connections with the wider Baltic world. Despite differences in culture and economies, the inland and coastal zones of the region were genetically homogenous until sometime post-1300 CE. After this, immigration from Sweden to the coastal zone caused a genetic shift that is still observed in the ancestry of modern coastal inhabitants. At the same time, the inland zone demonstrated ~ 1000 years of genetic continuity. These results shed light on the demographic context of Swedish political expansion into what is today Finland: they suggest that the consolidation of Swedish authority did not rely on widespread settlement by newcomers, but is instead consistent with historical interpretations that emphasise administrative, ecclesiastical, and sociopolitical integration. In line with archaeological evidence, we find that the population was growing rapidly with no evidence for widespread consanguinity. IBD sharing between Finnish individuals and other pre-modern individuals from around the Baltic Sea, along with archaeological evidence for long-distance connections, demonstrates that the inhabitants of pre-modern Finland were actively participating in wider networks of exchange and communication.

## Methods

### Sampling of human remains, laboratory work, sequence processing, and quality control

#### Archaeological sites

Permission for excavation and study of human remains was granted by the Finnish Heritage Agency. This study includes 38 individuals uncovered from 17 different archaeological sites in southwestern Finland. However, following 1240k target enrichment (see below), the genomes of four individuals did not yield reliable contamination estimates and, thus, were not included in analyses. Details of each site and burial are given in Additional file 3:

Text S1. For simplicity, we refer to sites by an abbreviation (see Fig. 1a—top-right inset for abbreviation key).

#### Laboratory sampling and experiments

For individuals from the sites EU1, EU2, HAK, HMH, HMM, HMP, HOS, JAN, KR1, MYI, PRK, PYL, RK2, RKA, RSK, TOM, and SIK, sampling of either tooth or bone, DNA extraction, and library preparation were carried out in the ancient DNA laboratory facility at the Max Planck Institute for Geoanthropology, Jena. For two individuals (KIR016, LUI019), libraries were prepared at the University of Tübingen. All libraries were sequenced at the Max Planck Institute for Evolutionary Anthropology (MPI-EVA), Leipzig. For individuals from the sites EU1, EU2, HMP, MYI, PRK, PYL, SIK, and two individuals from TOM (TOM002_ss, TOM003_ss), we prepared single-strand DNA libraries. Bone powder was drilled from either teeth or bone and DNA extracted with a silica-based method optimized for the recovery of short DNA fragments [73]. Briefly, lysates were prepared by adding 1 ml of extraction buffer (0.45 M EDTA, pH 8.0, 0.25 mg/ml proteinase K, 0.05% Tween-20) to the sample material in 2.0-ml Eppendorf Lo-Bind tubes and rotating the tubes at 37°C for approximately 16 h [74]). DNA was extracted using an automated liquid handling system (Bravo NGS Workstation B, Agilent Technologies). DNA was purified from 150 µl lysate using silica-coated magnetic beads and binding buffer D [74]. Extracts were converted to single-stranded libraries using an automated protocol [75, 76]. Libraries were prepared from 30 ml of extract, and library yield and efficiency were determined with quantitative PCR assays. The libraries were amplified and each library was tagged with a unique pair of indices using AccuPrime Pfx DNA polymerase. Libraries were sequenced to 5 million reads on Illumina HiSeq 4000 using 75 single-end cycles to screen their DNA preservation. All samples had ≥ 0.1% of sequences of ≥ 30 base pairs (bp) in length mapping to the human genome so were further enriched for human DNA with in-solution hybridization capture using capture probes that target approximately 1.2 million genome-wide ancestry-informative markers [77, 78]. The enriched libraries were then sequenced to approximately 20 million reads with the same platform and setup as above.

For individuals from the sites HAK, HMH, HMM, HOS, JAN, KIR, KR1, LUI, RK2, RKA, RSK, and TOM001, TOM003, TOM004, TOM005, TOM006, either bone or tooth powder was drilled and DNA extraction and library preparation were conducted manually as per [73]. Double-strand DNA libraries were prepared manually [79] and exposed to a partial uracil-N-glycosylase treatment to reduce the levels of DNA damage [80]. The libraries were enriched as described for single-strand libraries, and sequenced to approximately 20 million reads on Illumina HiSeq 4000 with 75 single-end cycles.

#### Bioinformatic processing

1. Adapter removal, mapping, pseudohaploid genotype calling
  Sequences were assigned to libraries based on index sequence, allowing for up to one mismatch in each index. Adapters were removed and forward and reverse reads merged using leeHom [81] (version: 1.1.5; parameters: –ancientdna). Sequences were mapped to the human reference genome (hg19) with BWA [82] (version: 0.7.12; parameters: -n 0.01 -o 2 -l 16,500). Mapped reads were processed using nf-core/eager versions: 2.4.5 and 2.5.0 [83], using the command: nextflow run nf-core/eager -r version -profile eva,archgen,medium_data,autorun,local_paths, TF -c https://github.com/MPI-EVA-Archaeogenetics/Autorun_eager/blob/main/conf/Autorun.config. Data were filtered to exclude reads shorter than 30 bp, prior to using picard MarkDuplicates v2.26.0 to remove PCR duplicates from each library. Where applicable, the unique reads from the captured double-stranded libraries were then trimmed to remove characteristic ancient DNA damage using bamutils v1.0.15 [84], removing the first and last 2 bp of all UDG-half treated reads. Single-stranded libraries were left untrimmed. The resulting mapped reads from each sample across libraries were then merged (where applicable). Pseudohaploid genotypes were called using pileupCaller v1.5.2, using the "randomHaploid" calling method, applying map and base quality filters, and deactivating per-Base-Alignment-Quality (parameters: -Q 30 -q 25 -B –randomHaploid). Single-stranded libraries were genotyped with the additional option –singleStrandMode. The 38 genomes displayed on-target coverage ranging from 0.0298X to 2.6459X (Additional file 1: Table S1).
2. Genetic sex determination
  Genetic sex determination was conducted using Sex.DetERRmine v1.1.2 [8] on reads filtered for map and base quality using samtools v1.12 with parameters -q30 -Q30. 19 individuals had a genetic sex of XX and 19 of XY (Additional file 1: Table S13).
3. Genotype likelihoods
  Each library's reads were divided into (1) reads shorter than the number of sequencing cycles and (2) reads equal or longer than the number of sequencing cycles using ATLAS’s “splitMerge” tool. The library's details were specified with "readGroupSettings" and the option "allowForLarger" was also employed. PMD scores for each library were estimated with ATLAS's "PMD" tool, as a function of distance from both ends for reads shorter than the number of sequencing cycles for double paired-end double-stranded libraries, and as a function of distance from the beginning of the read in case of single-stranded libraries. Minimum base quality was set at 30 and maximum length of estimation from the ends was set to 70. The human reference genome (hg19) was specified with the "fasta = " option. ATLAS's "recal" tool was used to reduce possible sequencing and reference biases. All the read groups from the samples' sequencing plate were pooled with the option "poolReadGroups" and the invariant position for the model was specified through the "regions" option, using a list of 10 million highly conserved genomic positions across 88 mammalian species downloaded from ensembl (https://grch37.ensembl.org/). The empiric PMD score calculation produced by the previous step was included in the recal calculation through the option "pmdFile" and the analysis was limited to chromosome 1 for computational reasons [85]. Genotypes on the autosomal chromosomes were called using ATLAS's "call" tool using the entire 1000 Genomes Phase 3 (1KGP) reference panel, using an "MLE" algorithm. PMD empiric pattern and recal patterns were included in the calculations with the "pmdFile" and "recal" flags respectively. The reference fasta file of the human reference genome (hg19) was specified with the "fasta" option, and the "printAll" flag was employed. Genotype calling was performed on bases with score equal or higher than 30 with the option "minQual = 30".
4. Imputation
  Imputation of autosomal genotypes was conducted via an internal pipeline that runs on the MPI-EVA cluster. This pipeline automatically imputes all individuals in an internal database of sequence data produced at MPI-EVA. Imputation was done using the entire 1KGP reference panel with GLIMPSEv1.1.1 [86], using default parameters and sex-averaged genetic maps from HapMap [87]. First, bcftools was used to generate a pileup from individuals’ bam files, with both minimum mapping quality and minimum base quality flags set to 30. The function GLIMPSE_phase was used to perform simultaneous imputation and phasing on genomic chunks of 2,000,000 bp with a buffer of 200,000 bp. We then used the integrated GLIMPSE_ligate and GLIMPSE_sample (with the flag –solve) functions and bcftools v1.387.91 [88] to obtain the final phased and imputed vcf files with the genotypes’ posterior probabilities (GP) at every 1KGP panel position. Imputed genotypes were then down-sampled to 1240k regions for haplotype modeling. We retained only those individuals’ genomes for whom the posterior genotype probability (GP) was ≥ 0.99 on at least 50% of the 1240k positions, or corresponding approximately with 400,000 pseudohaploid SNPs. ~ 400,000 is the minimum number of SNPs required to reliably run both *ancIBD* and *TTN*_*e*_. Following removal of a potentially contaminated individual (HMP001_ss), 22 of the newly presented genomes were of sufficient post-imputation quality for haplotype-based analyses (EU1002_ss, EU2001_ss, HMH001, HOS001, KIR016, LUI019, MYI001_ss, MYI002_ss, MYI003_ss, MYI004_ss, PRK004_ss, PYL003_ss, RK2002, RK2004, RK2009, RSK001, TOM001, TOM002, TOM003_ss, TOM004, TOM005, TOM006). In addition to our own data, the genomic data from 457 previously published ancient individuals were imputed as described above. This allowed for a thorough exploration of shared Identity-By-Descent (IBD) segments between the individuals presented in this study and previously published ancient individuals from around the Baltic and North Sea regions [3, 5, 10, 89, 90].
5. Uniparental haplogroups
  We genotyped the Y-chromosome reads using a Y-SNP list from the ISOGG (International Society of Genetic Genealogy) dataset included in the 1240k and Y-capture probes and by using an in-house script following the method described in [91]. We called pileups for all sites on the ISOGG list version 15.73, ignoring C-T and G-A transitions in the forward and reverse strands, respectively for single-strand libraries. This procedure allowed us to check in a semi-automatic way which positions were covered to assign an ancestral or derived haplogroup or to make corrections to calls in cases where, for instance, a more derived haplogroup was called because of residual ancient damage (C-T or G-A mismatches) in terminal read positions at diagnostic SNPs (Additional file 1: Table S1). The consensus mitochondrial sequence from *contamMix* for each individual was compiled into a single fasta file and mitochondrial haplogroups were called using *haplogrep3* [92] based on the mtDNA phylogeny (phylotree-rcrs@17.2). We only consider mitochondrial calls if their quality was greater or equal to 0.9 (Additional file 1: Table S1).
6. Contamination
  We used either DamageProfiler v0.4.9 or mapDamage2 v2.2.1 [93] to estimate authenticity of the extracted DNA by measuring the rate of C-to-T substitutions in the 5’ ends of the sequencing reads, and G-to-A substitutions in the 3’ ends (Additional file 1: Table S1). *contamMix* v1.0–10 [94] with the parameter –trim_bases 2 was used to estimate mitochondrial contamination (Additional file 1: Table S1). For genetic males, autosomal contamination was estimated using *ANGSD* v0.935 (Additional file 1: Table S1, [95]. The mtDNA-to-nuclear DNA (mt/nc) ratio for each individual was estimated using *mtnucratio* v0.7 (Additional file 1: Table S1). Using the conventional 5% modern contamination limit (estimated by *ANGSD* and/or *contamMix*), as well as qualitative markers of potential contamination (PCA shift, unusual sex chromosome ratios, inconsistency in Y chromosomal haplogroup markers, and damage patterns), we determined if an individual’s genome was appropriate for analysis. We excluded four individuals’ (PRK003_ss, HAK001, KR1002, EU2002_ss) genomes as a precaution because we could not derive a reliable estimate of contamination for them: (1) The genome of PRK003_ss displayed a nuclear contamination estimate of 28.19%. However, this estimate was based on only 23 SNPs. Contamination estimates from mtDNA could not be used as a proxy for nuclear contamination as the *mt/nc* ratio was far greater than 200 (*mt/nc* = 1318.24, see [96]). When calling Y-chromosomal haplogroups, several ancestral hits appeared among the derived hits (a sign of potential contamination); (2) The genome of HAK001 displayed an XX karyotype so nuclear contamination could not be determined directly. While the mtDNA contamination estimate was low, the *mt/nc* ratio was very high (*mt/nc* = 13,854.27). Given the low breadth of coverage (31,394 SNPs), coupled with a lack of reliable contamination estimate, we chose to exclude the genome of HAK001 from analyses; (3) The genome of KR1002 displayed an XX karyotype so nuclear contamination could not be determined directly. The genome only had 21,761 SNPs post-capture and an insufficient number of mitochondrial sequences for *ContamMix* analysis. Therefore, we excluded KR1002’s genome from analyses; (4) The genome of EU2002_ss displayed an XY karyotype. Both mtDNA and nuclear contamination estimates were based on very few SNPs (31 and 12, respectively, and *ContamMix* did not converge).
  We flagged the genome of one other individual (HMP001_ss) as potentially contaminated (mtDNA contamination > 5%) and so unsuitable for group-based analyses such as *f*-statistics but suitable for PCA projection. HMP001_ss was a single-strand library so the relatively high upper limit for mtDNA contamination could be either genuine contamination or the *contamMix* algorithm conflating features of a single-strand library (read length, damage patterns) with contamination. We considered the genome suitable for PCA projection but did not include it in grouped analyses due to the number of SNPs post-capture (*n* = 722,499). The remaining individuals were reliably estimated to have < 5% modern contamination.

### Population genetic analyses

The remaining 34 non-contaminated ancient genomes were merged with other publicly available modern and ancient genomes (Additional file 1: Table S14) stored in the Poseidon framework’s Community, Minotaur, and AADR archives [3–6, 8–12, 72, 78, 89, 90, 97–112]. Genomes were fetched and merged using Poseidon’s trident tool. We further merged these data with the same reference genomic datasets for present-day Finland as used in [10]. Briefly, these data consist of either Human Core Exome array data (THL_HCE) or whole-genome sequence (THL_WGS) data for individuals from the 1992, 1997, 2002, 2007, and 2012 cohorts of the National FINRISK Study [24] and the Health 2000 study [113], and only included individuals where both parents were born in the same municipality. THL_HCE consisted of 4710 FINRISK individuals and 145,639 SNPs overlapping the 1240k positions (Additional file 1: Table S14). The data consisted of twelve fewer individuals than in [10] due to 11 individuals revoking permission for use of their genome in research, and removing one individual with a high proportion of Sámi-like ancestry. The THL_WGS data set consisted of whole-genome sequences produced in FINRISK and Health 2000 studies https://thl.fi/en/our-services/thl-biobank1). THL_WGS consisted of 278 individuals and 936,564 SNPs overlapping the 1240k positions (Additional file 1: Table S14).

#### Principal component analysis

We performed two PCAs using EIGENSOFT’s smartpca tool [114] to visualize the ancestry of the ancient Finnish individuals within two different contexts: select Eurasian populations and all of Finland. The Eurasian populations were chosen to emphasise variation due to Uralic, Slavic, and Baltic ancestry. Both PCAs used the following parameters: lsqproject: YES, numoutevec: 4, newshrink: YES, outliermode: 2. The Eurasian PCA used popsizelimit: 300 and the Finnish PCA used popsizelimit: 100.

For the Eurasian PCA, we filtered the merged ancient and modern genomes on their shared Human Origins positions. We used the following populations for spanning: Udmurt, Croatian, French, Gagauz, Saami, Spanish, Belarusian, Czech, Russian_Archangelsk_Krasnoborsky, Russian_Archangelsk_Leshukonsky, Russian_Archangelsk_Pinezhsky, Ukrainian, Ukrainian_North, Polish, Estonian, Hungarian, Karelian, Mordovian, Veps, Saami.WGA, Chuvash, Lithuanian, Norwegian, Orcadian, English, Icelandic [72, 97, 100, 101, 105], and THL_WGS. We then projected the ancient individuals from Late Iron Age and Early Medieval Finland onto this PCA space. Additionally, we also projected the 26 currently-published ancient individuals with ≥ 30,000 SNP coverage on the 1240k panel from Finland with genome-wide data [8–12]. Finally, we visualised present-day Finnish genetic variation on the same PCA by constructing and projecting a 100% geometric convex hull from the THL_WGS data (Fig. 1b). The convex hull represents the smallest possible Euclidean space that contains the first and second principal coordinates of individuals in the THL_WGS data. It was constructed using the *chull()* and *slice()* functions in R and projected using *ggplot()* [115, 116].

The all-Finland PCA (Additional file 2: Fig. S1) was spanned using present-day Finnish individuals from the THL_HCE dataset grouped by administrative region: Etelä-Karjala, Etelä-Pohjanmaa, Etelä-Savo, Kainuu, Kanta-Häme, Keski-Pohjanmaa, Keski-Suomi, Kymenlaakso, Lappi, Päijät-Häme, Pirkanmaa, Pohjanmaa, Pohjois-Karjala, Pohjois-Pohjanmaa, Pohjois-Savo, Satakunta, Uusimaa, and Varsinais-Suomi. Given the large number of present-day individuals in these PCAs, we used the THL_HCE data to construct and project 50% geometric convex hulls at the provincial-level onto the PCA, thus, more clearly contextualising the ancient individuals within present-day genetic variation. Hull construction and projection was the same as in the preceding paragraph. We again projected the LIA/EM individuals from Finland onto this PCA. Since only 22 of the 34 non-contaminated genomes were of imputation quality and none of the modern data were imputed, we chose to span the PCA using only the modern genomes and to project the ancient genomes. This decision was made to maintain consistency in how the ancient individuals’ genomes were analysed.

#### Relatedness

We estimated pairwise relatedness between all newly-generated ancient genomes, as well as temporally relevant individuals from [10], using both BREADR [117] and READv2 [118]. We used BREADR to explicitly demonstrate uncertainty in estimates of relatedness (Additional file 1: Table S15). However, BREADR does not distinguish between parent–offspring and sibling-sibling pairs so we subsequently ran READv2 to determine these (Additional file 1: Table S16). We only considered 3rd-degree READv2 calls where the *z*-score associated with the pairwise mismatch rate between two individuals was greater than three (*Z* > 3, where *Z* is the number of standard errors the normalized mean P0 score is from a lower degree of relatedness). We found seven first-degree pairwise relationships (parent-offspring: RK2003 and RK2006; parent-offspring: MYI003_ss and MYI001_ss, MYI003_ss and MYI002_ss, MYI003_ss and MYI004_ss; siblings: MYI001_ss and MYI002_ss, MYI002_ss and MYI004_ss, MYI001_ss and MYI004_ss).

#### f_3_ and f_4_ statistics

We used xerxes v1.0.1.1 [98] to calculate individual F_3_statistics of the form F_3_(Modern_reference, Individual; Mbuti.DG) where Modern_reference is one of 154 present-day populations [72, 97, 99–101, 105, 119] (Additional file 1: Table S2, S14), and Individual is one of the 34 newly-sequenced ancient Finnish individuals from this study (the contaminated genomes of HAK001, KR1002, PRK003_ss, and EU2002_ss were not analysed any further). We used these F_3_ results, coupled with calibrated ^*14*^*C* and contextual dating, to define three temporal groups for estimating further *f*-statistics: LIA (*n* = 2), EM (*n* = 26), and PRK001_Outlier (*n* = 1) (see Additional file 1: Table S1 “Temporal_group_fstats”, “Geographical_group_fstats”, and “Coastal_Inland_ancient_fstats” for each individual’s per-analysis grouping). Note, the above group sizes account for the fact that we also removed the individual HMP001_ss that was deemed unsuitable for allele-based group analysis and the lower-coverage individual from each related pair (RK2006, MYI002_ss, MYI003_ss, MYI004_ss) from downstream analyses.

We estimated outgroup F_3_ statistics of the form F_3_(Modern_reference/Finnish_region, Analysis_group; Mbuti.DG) where Modern_reference is defined as above, Finnish_region is one of four regions (Southwest, East, North, Kainuu) into which we grouped the 18 modern Finnish administrative regions (THL_WGS dataset; see Additional file 1: Table S2), and the *f*_*3*_-statistic calculated was F3vanilla. We chose to analyze individuals from Kainuu as their own region due to the area’s modern-day inhabitants displaying greater genetic drift relative to the rest of the present-day Finnish population (see PCA position of Kainuu individuals in Additional file 2: Fig. S1). Among the global reference populations, F_3_ tests demonstrated that LIA and EM individuals shared most alleles with Karelians, Veps, Estonians, Finns (all Uralic-speaking), Lithuanians (geographically proximate), and Russians from Pinezhsky in Arkhangelsk (an area whose population was likely Uralic-speaking until the early medieval period [15]). The outlier PRK001_ss shared most alleles with Spanish, Lithuanian, Estonian, Norwegian, and Karelian. Using these results, we then tested if our analysis clusters were cladal with any of the five populations they shared most alleles with (TOP5) or any of the four Finnish regions. We specified F_4-_statistics of the form F_4_(Mbuti.DG, Modern_reference; Analysis_group, TOP5/Finnish_region).

To test whether the cultural, economic, and political divide between the southwestern coastal zone and the inland Häme region was also present as a genetic difference, we calculated F_4_-statistics between individuals grouped by which zone they were buried in. The coastal group, Coastal_ancient, consisted of EU2001_ss, EU1002_ss, PYL001_ss, PYL003_ss, PYL004_ss, PYL006_ss, RK2002, RK2003, RK2004, RK2007, RK2009, RKA001, SIK001_ss, and LUI019. The inland Häme group, Inland_ancient, consisted of HMH001, HMM001, HOS001, JAN001, KIR016, MYI001_ss, PRK004_ss, RSK001, TOM001, TOM002_ss, TOM003_ss, TOM004, TOM005, and TOM006. Despite being within the coastal zone, the sites at Eura Vanha Kansakoulu Kaunismäki and Eura Luistari are geographically intermediate between both zones. Therefore, we also calculated more conservative F_4_ statistics where the coastal group did not include EU2001_ss, EU1002_ss, nor LUI019 (Coastal_Strict). Since initial results suggested very similar ancestry profiles for the two LIA individuals (RSK001 and EU1002_ss), we included both of them alongside their EM counterparts in the inland and coastal groups, respectively. We calculated F_4_-statistics of the form F4(Mbuti.DG, REF; Coastal_ancient/Coastal_Strict, Inland_ancient), where REF was one of 30 ancient and modern groups (Additional file 1: Table S4).

Finally, we tested if our analysis groups (LIA, EM, Coastal_ancient, Inland_ancient) shared more or less affinity with a source of Siberian ancestry than do modern southwestern Finns (SW_Finns). All Uralic-speaking groups (except Hungarians) share an ancestry component that is related to ancient and present-day populations in Siberia [106, 120] and may have spread westward to Fennoscandia and the Baltics alongside the Seima-Turbino phenomenon. We calculated F_4_-statistics of the form F_4_(Mbuti.DG, Krasnoyarsk_krai_BA; Analysis_group, SW_Finns), where Krasnoyarsk_krai_BA is the genome of a Bronze Age individual from Siberia [102] and SW_Finns consists of present-day individuals from the administrative regions of Pirkanmaa, Satakunta, Varsinais-Suomi, Kanta-Häme, Uusimaa, Päijät-Häme, Kymenlaakso, and Keski-Suomi. For comparison, we tested the affinity of several other modern and ancient groups (Additional file 2: Fig. S5, Additional file 1: Table S7). We also estimated individual-level Siberian affinity (Additional file 2: Fig. S9, Additional file 1: Table S17).

#### Ancestry modelling

We used ADMIXTURE [121] to qualitatively explore potential ancestry sources among the individuals whose newly-generated pseudohaploid genomes displayed greater than 50,000 on-target 1240k SNPs. The following populations and individuals were merged together using Poseidon’s trident forge command: Mbuti.DG, Karitiana.DG, Papuan.DG, Onge.DG, Mixe.DG, Han, Ulchi, Even, Mala.DG, Makrani, Brahui, Balochi, BedouinB, Kalash, Armenian, Cypriot, Greek, Croatian, Sardinian, Basque, Spanish, English, Scottish, Orcadian, Norwegian, Icelandic, Hungarian, Czech, Polish.DG, Ukrainian, Ukrainian_North, Belarusian, Lithuanian, Estonian, Finnish, Karelian, Veps, Mordovian., Russian_Archangelsk_Krasnoborsky, Russian_Archangelsk_Leshukonsky, Russian_Archangelsk_Pinezhsky, Chuvash, Udmurt, Mari.SG, Mansi, Selkup, Nganasan, Saami.DG, Saami.WGA, EU1002_ss, EU2001_ss, HMH001, HMP001_ss, HOS001, JAN001, KIR016, LUI019, MYI001_ss, MYI002_ss, MYI003_ss, MYI004_ss, PRK001_ss, PRK004_ss, PYL001_ss, PYL003_ss, PYL004_ss, PYL006_ss, RK2002, RK2003, RK2004, RK2006, RK2009, RKA001, RSK001, SIK001_ss, TOM001, TOM002_ss, TOM003_ss, TOM004, TOM005, TOM006, ANE, CHG, EEHG, EN_LBK, WEHG, Villabruna_HG, Russia_Krasnoyarsk_BA.SG, Vapriikki_post-medieval, Vapriikki_medieval, Finland_Levanluhta, Finland_Levanluhta_B (see Additional file 1: Tables S1 and S14). Merged genotypes were subset to those SNPs that overlapped with the Human Origins panel. We then used PLINK to generate a .map file using the –recode flag, apply a minor allele frequency filter of 0.01, calculate linkage disequilibrium with the –indep-pairwise flag, a window size of 200 kilobases, a variant count of 25, and a pairwise R-squared threshold of 0.4, and prune the dataset to include only unlinked variants. ADMIXTURE was run with a *kmin* of 2 and a *kmax* of 12, with each *k* run for five replicates. Estimation of the cross-validation error suggested best *k* ranged from 7–9. See Additional file 4: Fig. S10 and Additional file 1: Table S18.

We used *Admixtools2* [122] to formally model both the distal and proximal ancestry of the four analysis groups (LIA, EM, Coastal_ancient, Inland_ancient). Note, to increase power for ancestry modelling we expanded EM to include five individuals from [10] that overlap both geographically and temporally with the EM in Finland (PKN008_ss, PLN001_ss, PLN002_ss, PLN004_ss, and TMP003). For distal models, we implemented a rotational model with five potential sources (Russia_Krasnoyarsk_BA.SG, EEHG (Eastern European Hunter-Gatherer), WEHG (Western European Hunter-Gatherer), Steppe, EN_LBK, see Additional file 1: Table S14) and six outgroups (Ethiopia_4500BP.DG, CHG (Caucasus Hunter-Gatherer), Russia_CentralYakutia_LN.SG, ANE (Ancient North Eurasian), Villabruna_HG, Anatolian Neolithic Farmer, see Additional file 1: Table S14). The five sources represent the major ancestral components of most modern western European [6, 100, 123] and Uralic-speaking European populations [71]. Model rotation allowed us to compare all combinations of the five source populations for each target population. When a source was not used in a model, it was “rotated out” and moved to the list of outgroups for that particular model [124]. We then ran all potential, rotated models sequentially using *Admixtools2’s qpadm_multi()* function with allsnps = TRUE. We used this same framework to also model the ancestry of geographically proximate or linguistically related modern populations and geographically proximate ancient populations in order to compare them with our analysis groups. The modern comparison populations were SW_Finns (as defined above), Coastal_Modern (*n* = 12, THL_WGS individuals from the modern-day administrative regions of Varsinais-Suomi, Satakunta), Inland_Modern (*n* = 14, THL_WGS individuals from the modern-day administrative regions of Pirkanmaa, Kanta-Häme, Päijät-Häme), Sámi, Karelian, Veps, Mordovian, Mansi, Mari, Estonian, Nganasan, Norwegian, Russian_Archangelsk_Krasnoborsky, Russian_Archangelsk_Leshukonsky, and Russian_Archangelsk_Pinezhsky [72, 99–101]. Ancient comparison populations were Pirkanmaa_Post-Medieval [10], KUU001_ss [12], Russia_Bolshoy, Finland_Levanluhta, Finland_Levanluhta_B [8], Estonia_IA.SG, Russia_IA_Ingria.SG[106], Finland_Saami_IA.SG [9], Estonia_EarlyViking.SG [3], central_Sweden_Pre-Viking, central_Sweden_Late_Viking, south_Sweden_Pre-Viking, Gotland_Pre-Viking, Gotland_Viking, Gotland_Late_Viking [4]. We only considered feasible models where none of the ancestry components were negative. We then filtered the remaining models so that all *Z*-scores for each ancestry component satisfied |*Z*|≥ 2, and *p* ≥ 0.01, and if possible, twice the standard error for a component was less than 0.7. If this last condition was not met and only one model for a target passed the previous two conditions, we included that model. If two or more models passed for a target, we parsimoniously chose the model with fewer ancestry components. If a target had multiple passing models each with the same number of ancestry components, we chose the model whose standard error for the minor ancestry component was smaller. Similar to the studies of [120, 125–129], we used a *p*-value threshold of *p* ≥ 0.01 to assess our models due to the scarcity of the ancient DNA record from the Baltic region, especially modern-day Finland, and to ensure model consistency across individuals/groups. As discussed in [130], a more relaxed *p-*value threshold allows us to retain biologically/historically meaningful models even when the source populations used in a particular model set are not ideal proxies for the true, but unknown, sources. Furthermore, *p*-values in the range of 0.01–0.05 can be often manipulated by selecting different right populations, but this renders models across targets difficult to compare. Thus, we prioritised model consistency (same right populations, same sources) rather than adhering to a strict *p*-value threshold.

For proximal models, we sought to model LIA, EM, Coastal_ancient, Coastal_Modern, Inland_ancient, Inland_Modern, and SW_Finns as a mixture of Baltic, Scandinavian, and Sámi-related ancestries. We reviewed the literature and selected potential proximal sources and divided them into three categories: Scandinavian, Baltic, and Saamic. All potential sources except present-day Sámi predate our targets. Saamic sources included Saami.DG, Russia_Bolshoy, and Finland_Levanluhta; Baltic sources included Russia_IA_Ingria.SG and Estonia_IA.SG; and Scandinavian sources included central_Sweden_Pre-Viking, central_Sweden_Viking, north_Sweden_Pre-Viking, south_Sweden_Pre-Viking, Sweden_IA.SG, and Estonia_EarlyViking.SG [4, 8, 62, 100, 106, 119]. Despite being found in Estonia, the Viking Age individuals from Saaremaa in Estonia have been shown using qpAdm modelling to be genetically Scandinavian [3]. Additionally, their burial goods indicate that they most likely came from the Mälaren valley in modern-day Sweden [3]. Again, we implemented a rotational model scheme: we ran 3-way models that had one Baltic, one Scandinavian, and one Saamic source and all their constituent 1–2-way submodels using *Admixtools2's qpadm_multi()* and *rotate_models()* functions. We also modelled the ancestry of roughly contemporaneous Baltic and Scandinavian populations. We filtered models with the following criteria: the model was feasible, all *Z*-scores satisfied |*Z*|≥ 2, and *p*-value ≥ 0.01. Among acceptable models, we identified a three-population combination that provided an acceptable model for all targets. Two such combinations were found: south_Sweden_Pre-Viking + Estonia_IA.SG + Finland_Levanluhta and south_Sweden_Pre-Viking + Estonia_IA.SG + Saami.DG. We chose to show models with Finland_Levanluhta, because Saami.DG post-dates the ancient targets. See Additional file 1: Tables S5, S6.

The ancestry of Coastal_ancient was best modelled using a 3-way model of Scandinavian, Baltic Iron Age, and Saamic ancestry (49.22%, 35.8%, and 14.99%, respectively; Fig. 1b—inset, Additional file 1: Table S5). However, Coastal_Modern required only Scandinavian and Saamic sources (74.19% and 25.81%, respectively; Fig. 1b—inset, Additional file 1: Table S5). This result suggests a large genetic shift in the coastal population of southwestern Finland within the last ~ 700 years. Such a change would be in line with archaeological and historic records of increasing Swedish influence in the region as the medieval Kingdom of Sweden exerted its hegemony eastward (castle and town building, population movement). Indeed, many present-day Swedish-speaking Finns (*Finlandssvenskar*) live in the southwestern coastal zone and the culture is heavily influenced from Scandinavia.

Since this would be the first time genomic (ancient or modern) data has been used to show such a historically important population shift in Finland, we sought to test the robustness of the result. First, we tested if the result was sensitive to the choice of individuals chosen to proxy Baltic ancestry (Estonia_IA [106]). We included contemporaneous medieval Estonian genomes (Estonian_Medieval.SG: OLS3, Ila, Ilg) from [106] and reran the proximal qpAdm models with Estonian_Medieval.SG instead of Estonia_IA among the Baltic sources. The results were unchanged and the best-fitting model for Coastal_Modern still did not include a source of Baltic ancestry (Additional file 1: Table S21). Next, we ran a series of one-way proximal models to test the hypothesis that the Coastal_Modern population is a direct continuation of the Coastal_ancient population. We set Ethiopia_4500BP.DG as a true outgroup but also included the groups Finland_Levanluhta, Estonia_IA.SG, and south_Sweden_Pre-Viking among the right populations. First, we ran this model with all four right populations, and then dropped south_Sweden_Pre-Viking from the right populations. The first model failed (*p* < 0.01, Additional file 1: Table S21) but the second model without a source of Scandinavian ancestry among the right populations passed (*p* > 0.01, Additional file 1: Table S21). Including the Scandinavian source among the right populations “broke” the model (i.e., the *p*-value dropped below 0.01), strongly indicating differential affinity with Scandinavian ancestry for the Coastal_Modern group relative to Coastal_ancient.

The apparent increase in Steppe ancestry between ancient and modern Finnish groups (Additional file 2: Fig. S11) was investigated in order to determine if such a systematic change represented a true signal. Further investigation was warranted since we could think of no known archaeological or genetic evidence for why such an increase might exist. We ran F_4_-statistics of the form F_4_(Mbuti.DG, WEHG/Steppe; SW_Finns/Coastal_modern/Inland_modern,Early_Medieval_Finland/Late_Iron_Age_Finland/Coastal_ancient/Inland_ancient) using (1) all 1240k positions and (2) using the 936,564 SNPs from the THL_WGS dataset (which tests for bias due to comparing capture and WGS data). For both sets of SNPs, we found that Early Medieval Finland and Coastal_ancient displayed evidence for increased geneflow with both WEHG and Steppe (Additional file 1: Table S22). Therefore, since the qpAdm signal has no known historical explanation, coupled with the signal not being due to a capture-WGS bias, we believe it to be a case of “ancient attraction” where reference bias and correlated patterns of DNA damage cause ancient populations to appear to share more alleles with each other than is actually the case [131, 132].

#### Demography

##### hapROH

Using pseudohaploid data, we inferred runs of homozygosity (ROH) for each LIA and EM individual with ≥ 400,000 SNPs (EU1002_ss, HMH001, HOS001, KIR016, LUI019, MYI001_ss, PRK004_ss, RK2002, RK2004, RK2009, RSK001, TOM001, TOM003_ss, TOM004, TOM005, TOM006), using the *hapROH* Python package [133]. In order to better resolve population dynamics, we included five individuals from [10] that overlapped both geographically and temporally with the Early Medieval Period in Finland (PKN008_ss, PLN001_ss, PLN002_ss, PLN004_ss, TMP003). See Additional file 1: Table S12.

##### ancIBD

We ran *ancIBD* [134] to study Identity-By-Descent (IBD) segment sharing between 22 ancient individuals with imputed genomes (see Imputation subsection above) presented in this study and the imputed genomes of 457 previously published ancient individuals from around the Baltic and North Sea regions [3–5, 10, 135, 136] from the pre-Viking Age to the Early Modern Period (Additional file 1: Table S9). The previously published individuals come from diverse genetic and cultural backgrounds—Anglo-Saxon and Germanic groups [4, 136]; a highly genetically diverse group of individuals associated with the cosmopolitanism of the Viking Age (3); early Slavic communities [5, 135]; and an agricultural community from Medieval/Early Modern Finland [10]. We restricted the analysis to individuals dating from the Viking Age (700–1050 CE) to the Early Modern Period (1500–1850 CE) whose post-imputation quality was high enough to confidently use *ancIBD*. IBD segments ≥ 8 cM were called between individuals. To reduce the rate of false-positives, we removed pairs that shared only a single IBD connection. In order to capture patterns of movement rather than general background relatedness due to shared common ancestry, we further filtered to include only individuals that shared long IBDs (≥ 16 cM). For pairs of individuals only connected by IBD segments ≤ 12 cM, we required the average maxGP(0.99) to be > 0.7. Since we specifically wanted to study circum-Baltic patterns of IBD sharing, we removed connections between individuals outside of the study area i.e., IBD connections between individuals from Sweden and Iceland. This resulted in 245 individuals and 379 IBD connections across nine regions (Denmark, Finland, Germany, Gotland, Latvia, Norway, Poland, Russia, Sweden). This resulted in one main network and multiple smaller satellite networks (Additional file 2: Fig. S8, Additional file 1: Table S9). Using Cytoscape [137], we applied a prefuse force directed layout algorithm to the initial visualisation of the entire network graph. This causes individuals that share more IBD segments to be pushed more closely together. This layout algorithm emphasises regional clusters in addition to highlighting those individuals in the network that connect larger clusters of individuals together. We removed satellite networks which left a final contiguous network for visualization that consisted of 103 individuals with 266 shared IBD links. Finally, we visualized the strength of connection between two individuals by scaling network edges by the total number of shared IBD segments between a pair of individuals (Fig. 3a).

Next, we used the IBD data from the individuals visualised in the main network and those visualized in the satellite networks to calculate average IBD sharing between regions (Fig. 3b, Additional file 1: Table S10). This was estimated by dividing the sum of IBD segment length (measured in centiMorgans) between two regions by the total number of potential pairwise relationships between those two regions (measured as the number of individuals in the network from that region as well as unconnected individuals). This allowed us to estimate the average rate of between-region IBD sharing, accounting for differences in sample size between regions.

##### TTNe

Using the shared IBD connections between individuals from southwestern Finland, we estimated the effective population size (*N*_*e*_) over time using *TTN*_*e*_. *TTN*_*e*_ applies coalescent modelling within a maximum likelihood framework to estimate *N*_*e*_ at the number of generations ago an individual within a time-series of such individuals lived [53]. *TTN*_*e*_ split individuals into the following time series: seven samples in age bin 0 (676–821 BP): [HOS001, LUI019, PKN002, PKN006, PKN008, RK2002, TOM001]; 16 samples in age bin 1 (821–966 BP): [EU2001, HMH001, MYI001, PLN001, PLN002, PLN003, PLN004, PRK004, PYL003, RK2004, RK2009, TOM002, TOM003, TOM004, TOM005, TOM006]; and two samples in age bin 2 (966-1111BP): [EU1002, RSK001]. Only bins 0 and 1 were used for inference. To increase power, we incorporated eight Early Medieval individuals from [10]. *TTN*_*e*_ was run with the following flags: –minN 7 –Tmax 50, –minl_infer 8 –maxl_infer 20 –out output_all_iids –autocv –boot –np 10 (Additional file 1: Table S11). We adopt a conservative approach to inference and consider *N*_*e*_ estimates from only as far back as 21 generations ago (roughly corresponding to about 50 years before the upper estimate of the age of the oldest individual in this study [EU1002_ss]). This is done to avoid interpreting trends in *N*_*e*_ that may be the result of *TTN*_*e*_’s regularization scheme [53].

##### IBIS

We used *IBIS* [135] to identify shared IBD segments between the genomes of ancient individuals from southwestern Finland and present-day Finnish inhabitants. GLIMPSE was used to impute the new ancient individuals in this study whose genomes had ≥ 400,000 pseudohaploid SNP coverage and were not genetic outliers (RK2002, RK2004, RK2009, EU1002_ss, HOS001, HMH001, TOM001, TOM003, TOM004, TOM005, TOM006, MYI001, KIR016). This is roughly equivalent to 1X coverage on the 1240k SNP panel. Positions were filtered to include only those with posterior probability of 0.99 or higher. Plink was used to convert the filtered VCF file for each ancient genome to plink format using the following flags: –keep-allele-order, –snps-only, –double-id, –make-bed, –out. The ancient genomes were then individually merged with 4710 present-day Finns from the THL_HCE dataset using Poseidon’s trident forge command with the following flags: –intersect, –onlyGeno. Individual-level merging was done to prevent cumulative loss of data. The number of positions remaining post-merge ranged from 120,361 for TOM001 to 198,618 for HOS001 (see Additional file 1: Table S23 for all post-merge position counts). For each individual .bim file, we added the genetic map for build GRCh37 of the human reference genome using *IBIS’s* add-map-plink.pl command. We ran *IBIS* with the following flags: -mt 300, -er 0.004, -maxDist 0.1, and -min_l 8. We grouped individuals into coastal (RK2002, RK2004, RK2009, EU1002_ss) or inland (HOS001, HMH001, TOM001, TOM003_ss, TOM004, TOM005, TOM006, MYI001_ss, KIR016), calculated the pairwise sum IBD for each ancient-modern pair, and averaged these sums per present-day Finnish municipality. This yielded a “connectedness” score i.e., how much IBD an ancient group shared with any given municipality across Finland. We then used *gstat*’s kriging interpolation [115, 138] to visualise this genetic connectivity in R, implemented through RStudio [116, 139]. Following the method used in [10], we visualised both “common” and “rare” segment sharing between the groups of ancient individuals and present-day Finns. Common segments are defined as those IBD segments that are shared between one ancient and many modern individuals, while rare segments are defined as those IBD segments that an ancient individual shares with only one modern individual. We performed kriging at the group level (coastal or inland) in order to increase the resolution of region-specific IBD sharing “hotspots”. We fit variograms with a range of 200 km for common segments and a range of 150 km for rare segments. Software versions are given in Table S24.

## Supplementary Information

Additional file 1: Supplementary tables. Tables S1-S25.

Additional file 2: Supplementary figures. Figs. S1-S9, Fig. S10 caption, Fig. S11.

Additional file 3: Text S1. Archaeological details of studied individuals.

Additional file 4: Supplementary figure. Fig. S10 ADMIXTURE plot.

## Data Availability

Raw sequencing data from the newly sequenced individuals have been deposited at the European Nucleotide Archive (ENA) as ENA:PRJEB104645 and are publicly available [140]. Genotype data are available from the Poseidon community archive: https://github.com/poseidon-framework/community-archive [98]. Genotype data are also available from Zenodo: https://doi.org/10.5281/zenodo.21991261 [141]. This paper analyzes existing, publicly available genotype data, accessible at https://github.com/poseidon-framework/ [98]. This paper analyzes existing genotype data from THL Biobank, accessible via application procedure from THL Biobank https://thl.fi/en/our-services/thl-biobank1. This paper does not report original code.
