# Supplementary material for: Population genomics, demography, and circum-Baltic connectivity of Early Medieval southwestern Finland
Source: Genome Biol. 2026 Sep 15;27:288. doi: 10.1186/s13059-026-04277-7 (PMC13576218; doi:10.1186/s13059-026-04277-7)
Supplement: Supplementary file 1 [file 13059_2026_4277_MOESM2_ESM.pdf]

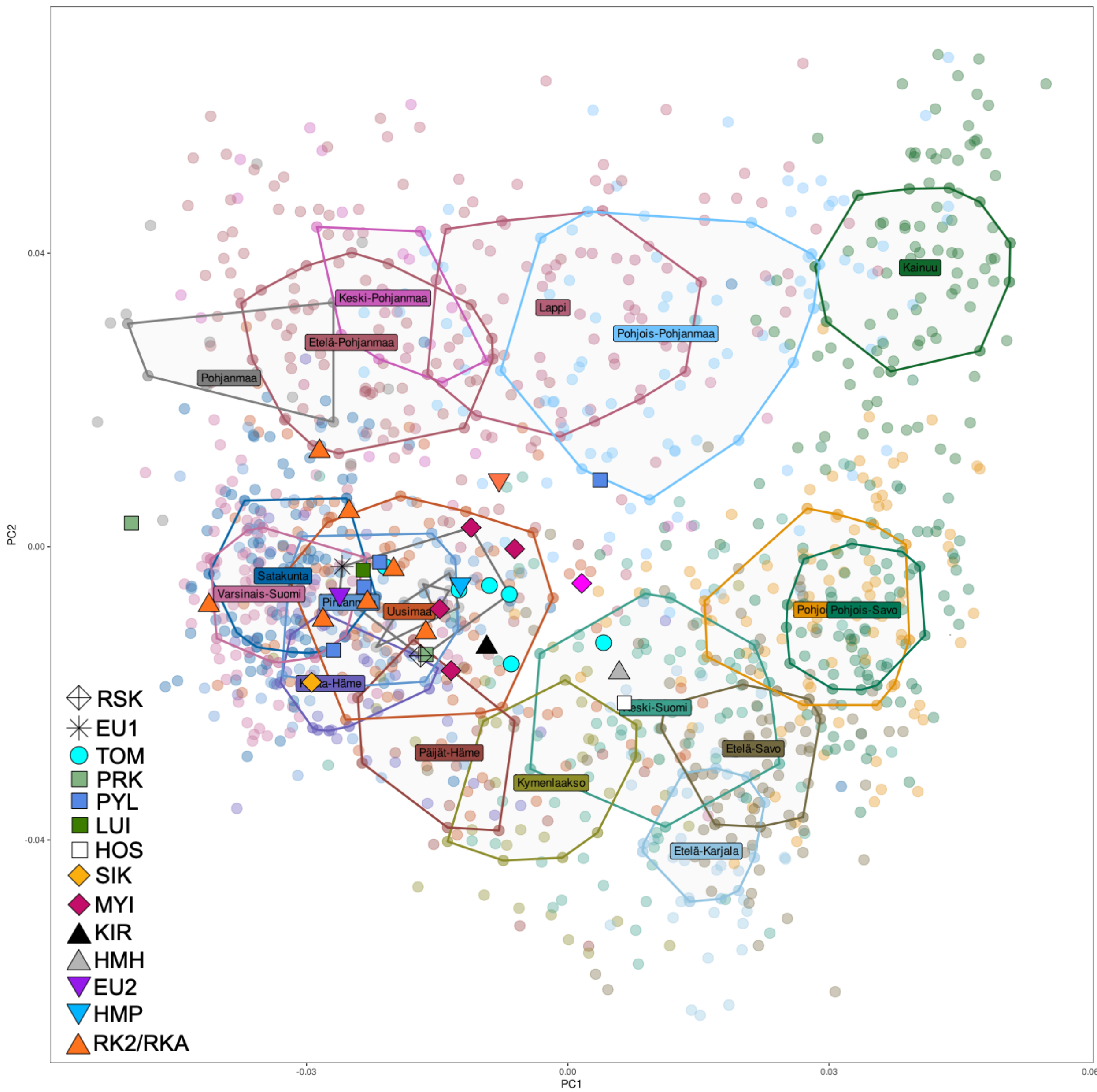

Fig. S1: PCA of present-day genetic variation in Finland with the 33 non-contaminated ancient genomes from this study projected. The PCA was spanned using present-day Finnish Biobank donors from the THL_HCE dataset, using a maximum of 100 individuals per administrative region. Administrative region labels lie on the median PC1 and PC2 coordinates for each administrative region and a 50% geometric hull is projected around each label in order to better visualise the core genetic variation of an administrative region. Late Iron Age individuals from Finland are represented using black-line symbols. Early Medieval Period individuals are represented using the coloured polygons. For clarity, only the first three characters of each individual's laboratory code are shown in the figure key.

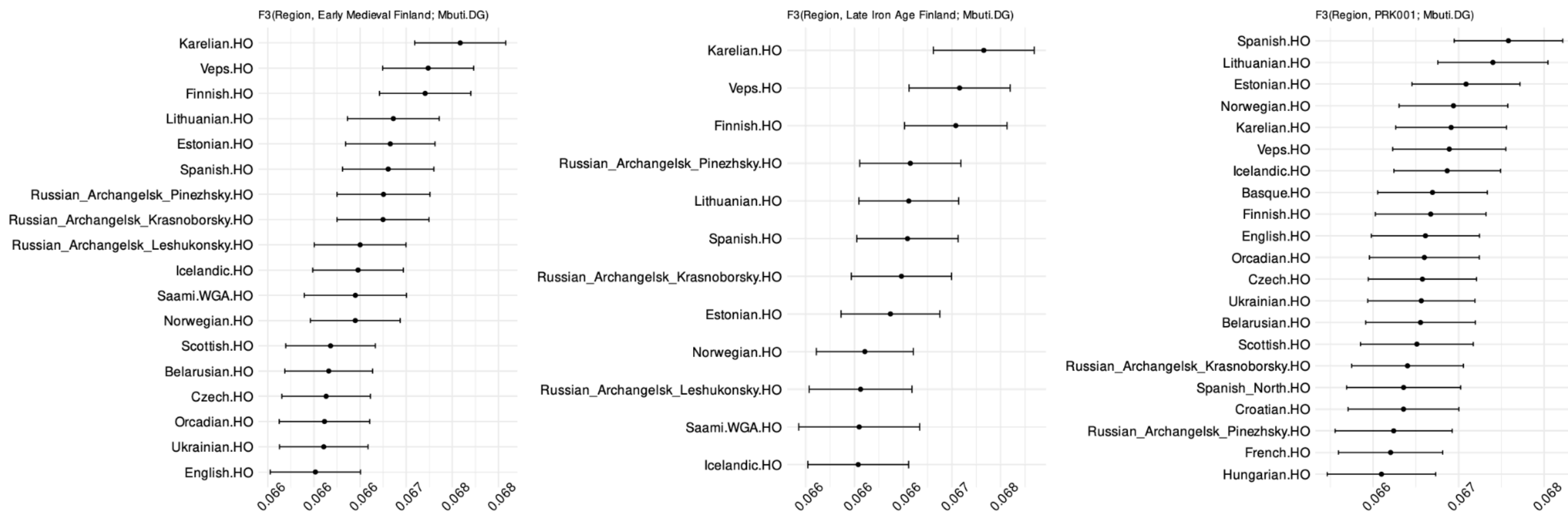

Fig. S2: f_3-statistics testing for similarity in allele frequencies between ancient individuals from the Late Iron Age and Early Medieval Finland and 154 modern reference populations. See Additional File 1: Table S2.

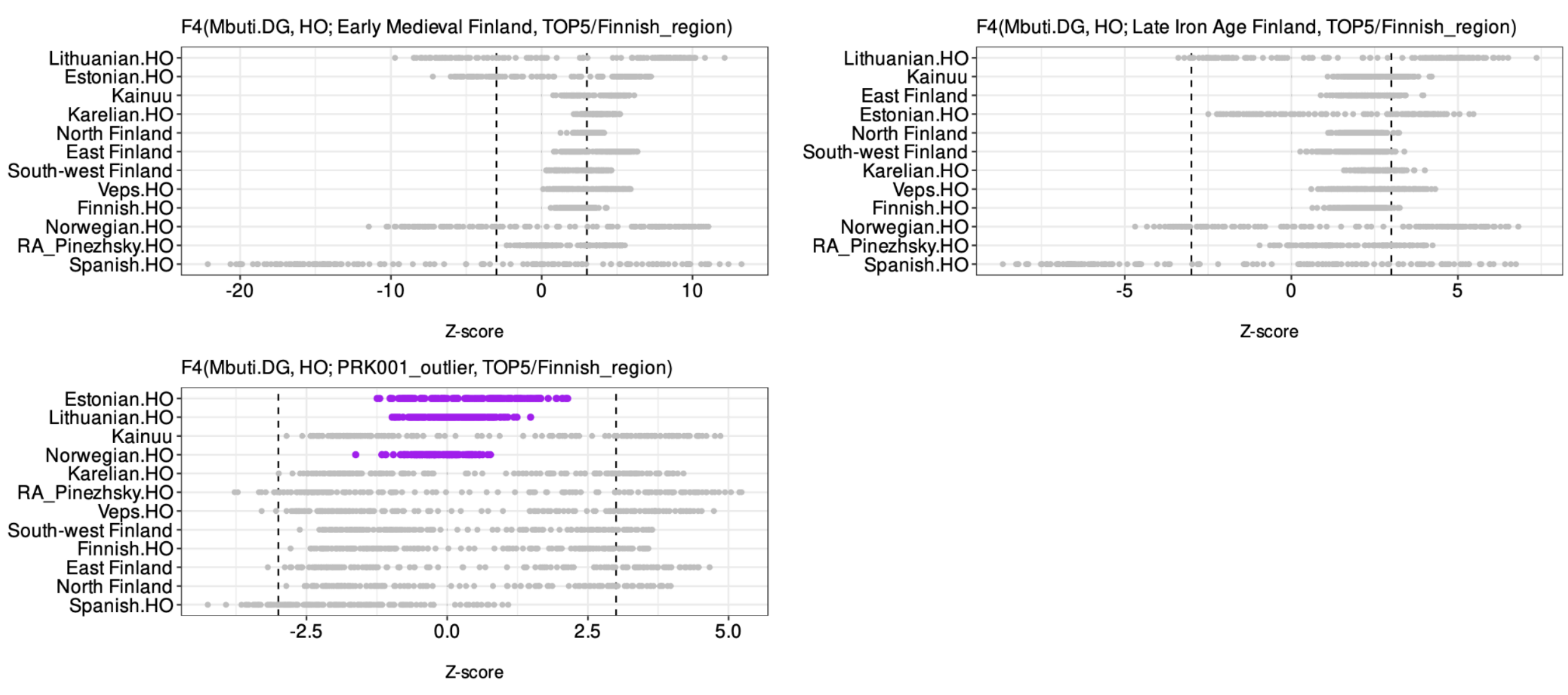

Fig. S3: f_4-statistics testing for cladality between ancient individuals from Late Iron Age and Early Medieval Finland with the four Finnish regions and the top five modern reference populations (HO) with which the ancient Finnish groups shared alleles. The vertical dashed lines indicate Z-scores where $|Z|>3$. Populations cladal with the test population/individual are shown in purple. RA_Pinezhsky.HO refers to individuals from

the Russian_Archangelsk_Pinezhsky group genotyped on the Human Origins array. See Additional File 1: Table S3.

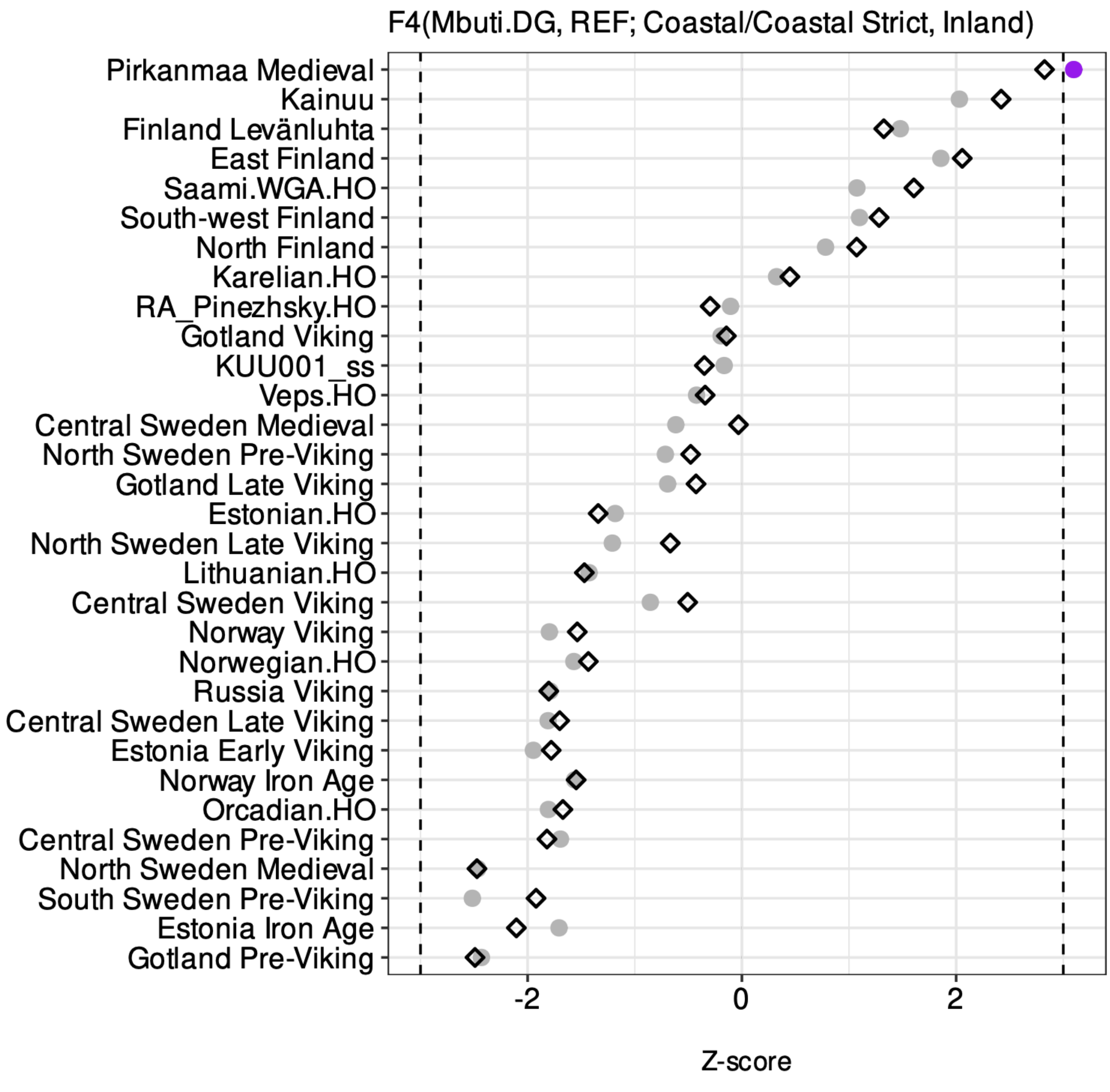

Fig. S4: f_4-statistics testing for cladality between ancient individuals from Coastal_ancient/Coastal Strict and Inland_ancient southwestern Finland. Circles represent the Z-scores for the test with the Coastal_ancient group, and hollow diamonds represent the Z-scores for the Coastal Strict group that did not include individuals from the intermediate sites of EU1/EU2, and LUI. The vertical dashed lines indicate Z-scores |Z|>3. RA_Pinezhsky.HO refers to individuals from the Russian_Archangelsk_Pinezhsky group genotyped on the Human Origins array. See Additional File 1: Table S4.

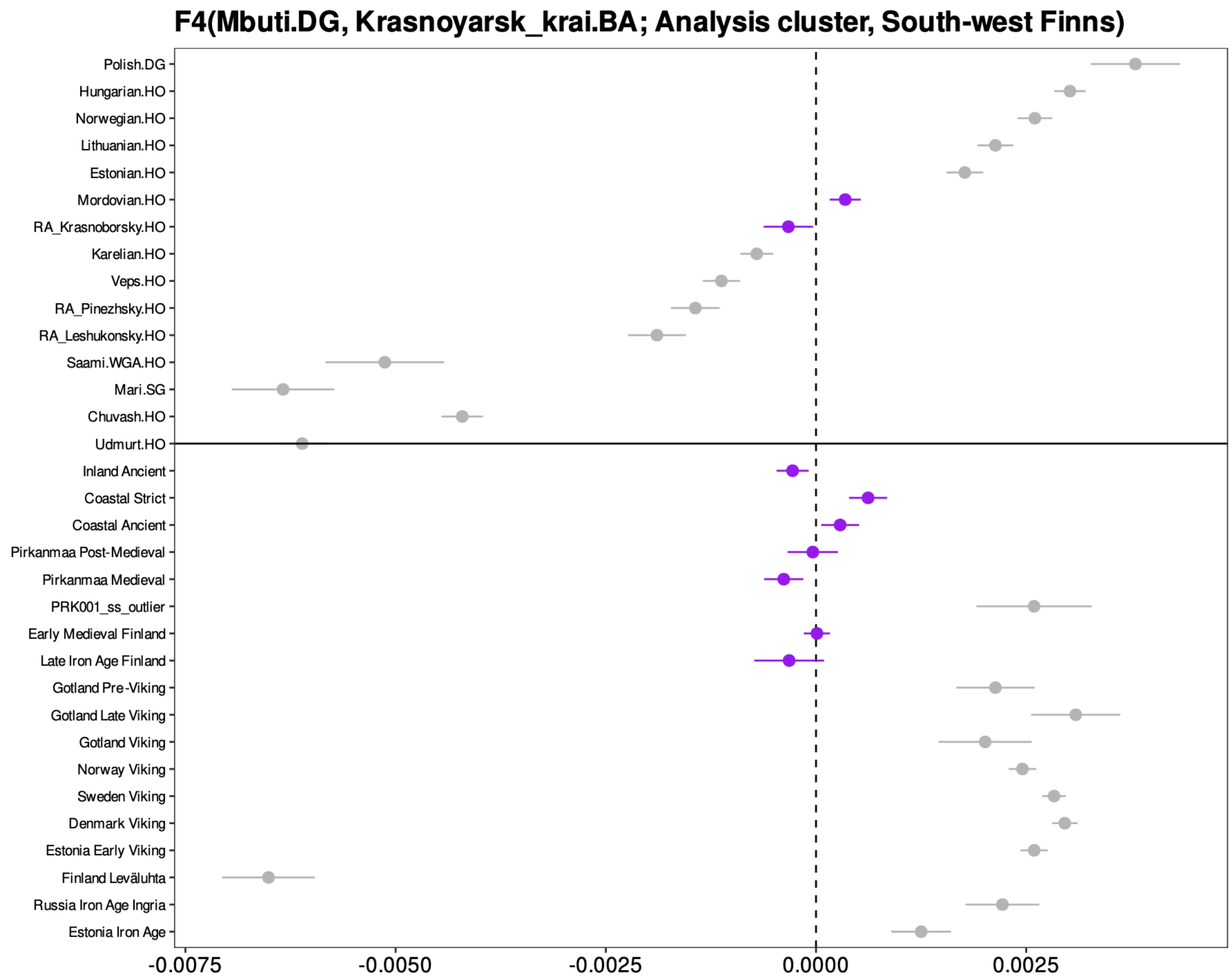

Fig. S5: f_4-stastistics for assessing excess allele sharing between ancient groups from southwestern Finland (Inland Ancient, Coastal Ancient, Coastal Strict, Late Iron Age, Early Medieval) and an ancient source of Siberian ancestry from Bronze Age Siberia (Krasnoyarsk_krai.BA), relative to present-day southwestern Finns. Increasingly negative values indicate greater affinity (i.e., allele sharing) with the ancient source relative to present-day southwestern Finns, and vice-versa. Previously published modern (above horizontal black line) and ancient (below horizontal black line) groups are shown for comparison. Purple indicates that a group did not display significantly different levels of allele sharing with the ancient source compared to present-day southwestern Finns ($|Z|<3$). RA_*.HO refers to individuals from the Russian_Archangelsk_Pinezhsky, Russian_Archangelsk_Krasnoborsky, and Russian_Archangelsk_Leshukonsky groups genotyped on the Human Origins array. See Additional File 1: Table S7.

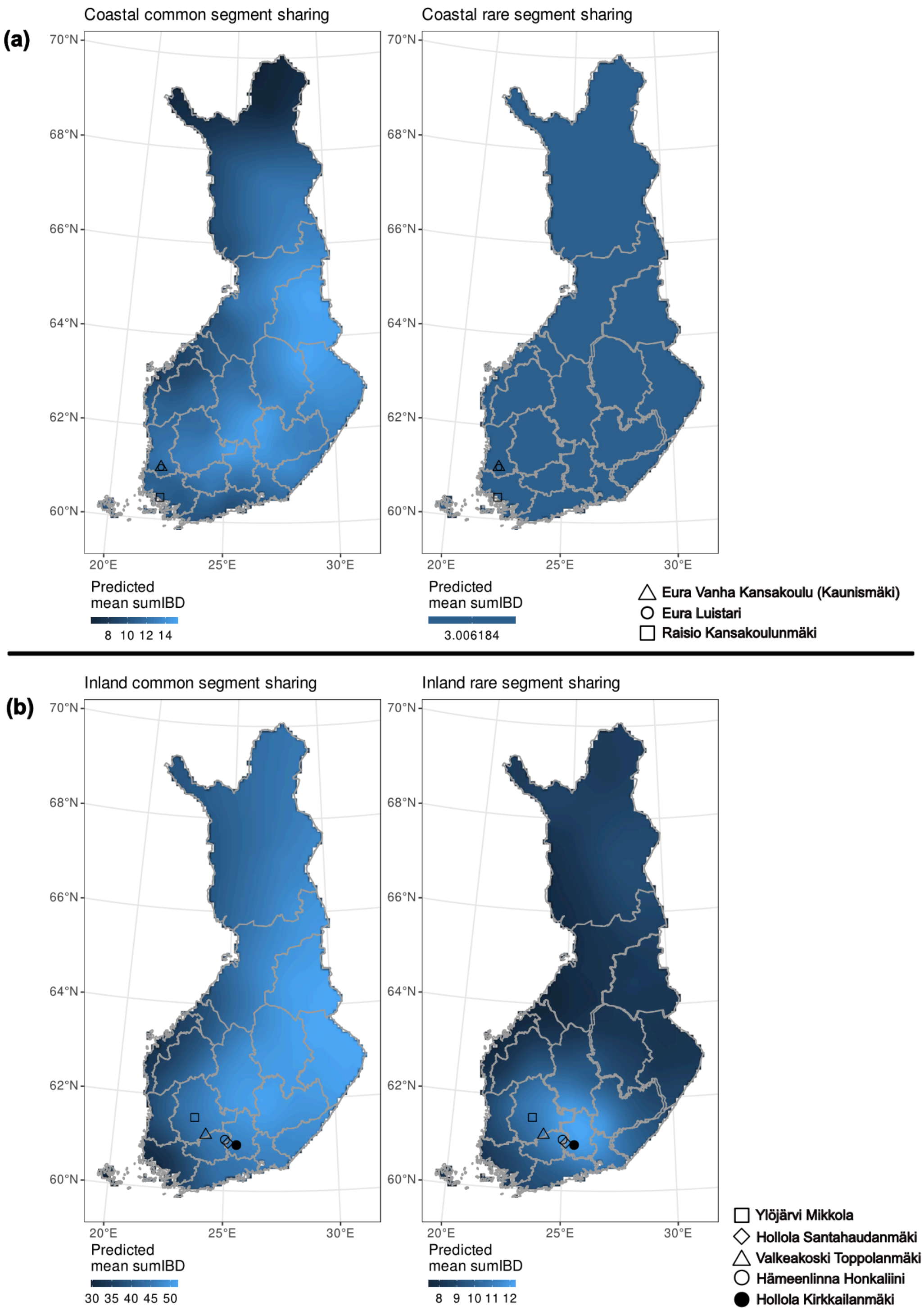
(a)
Coastal common segment sharing
Coastal rare segment sharing
70°N
68°N
66°N
64°N
62°N
60°N
20°E
25°E
30°E
Predicted mean sumIBD
8 10 12 14
Predicted mean sumIBD
3.006184
Eura Vanha Kansakoulu (Kaunismäki)
Eura Luistari
Raisio Kansakoulunmäki
(b)
Inland common segment sharing
Inland rare segment sharing
Predicted mean sumIBD
30 35 40 45 50
Predicted mean sumIBD
8 9 10 11 12
Ylöjärvi Mikkola
Hollola Santahaudanmäki
Valkeakoski Toppolanmäki
Hämeenlinna Honkaliini
Hollola Kirkkailanmäki

Fig. S6: Interpolated IBD connectedness between (a) ancient Coastal zone and (b) ancient Inland zone individuals and present-day Finnish THL Biobank donors. Normalized sums of IBD segment lengths between ancient-modern pairs are calculated at the level of the municipality separately for the Inland and Coastal zones. The municipality average of this figure is used for kriging. Lighter shading corresponds to higher IBD connectedness, and darker shading corresponds to lower IBD connectedness. Common segments refer to IBD segments that an ancient individual shares with more than one modern individual. Segments that an ancient individual shares with only one modern individual are here referred to as rare segments. Modern Finnish administrative region sample sizes are in Additional File 1: Table S14.

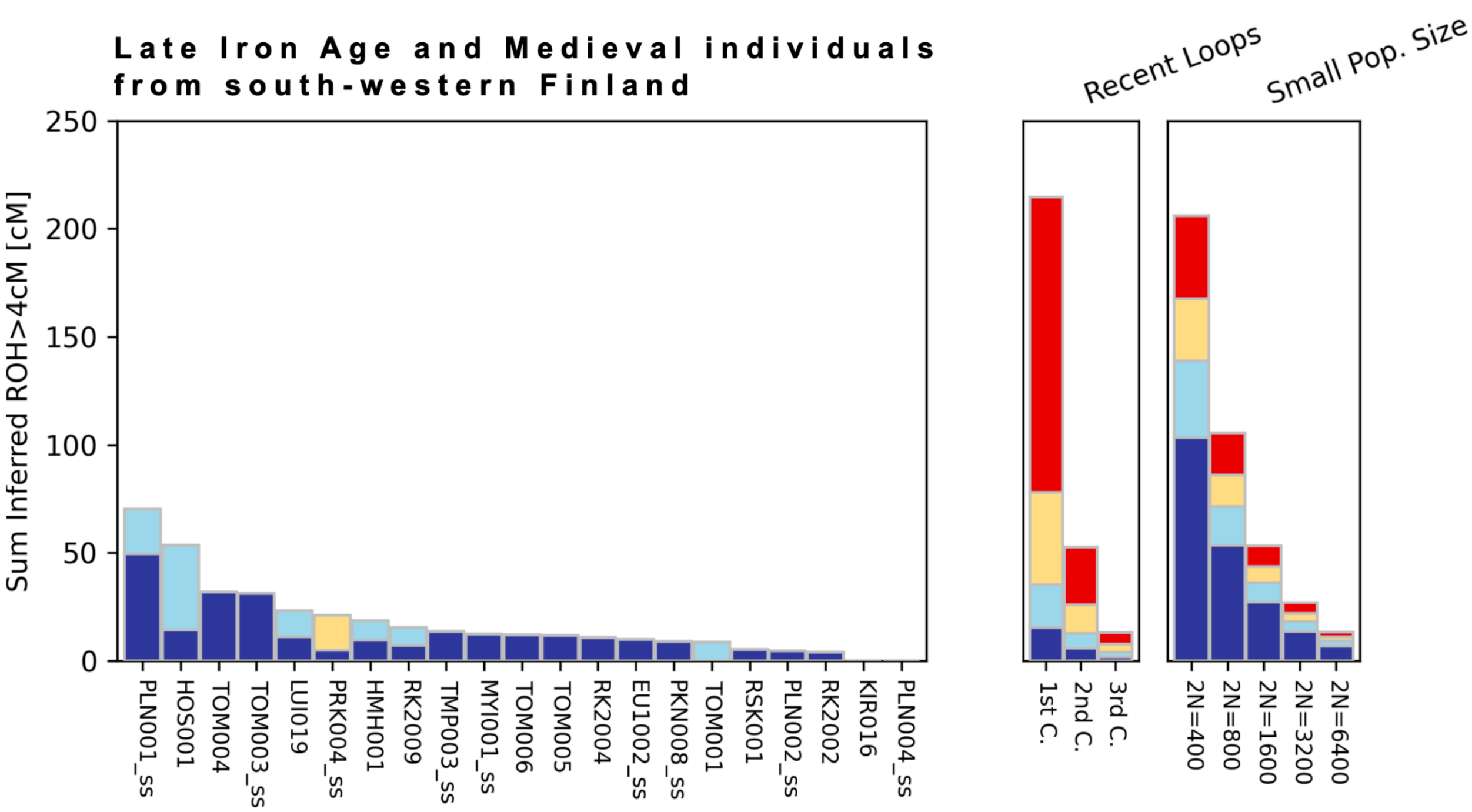

Fig. S7: Runs of Homozygosity (ROH) for Late Iron Age and Early Medieval individuals from southwestern Finland with genomes whose coverage was ≥400,000 pseudohaploid SNPs. Five temporally-overlapping individuals from (10) with sufficient coverage are included as well. Barplots are divided by colours which correspond to the sum of ROH within each length class (dark blue: 4-8 cM, light blue: 8-12 cM, yellow: 12-20 cM, red: >20 cM). Rightmost panels are the predicted ROH for individuals whose parents are closely related (1st C. = 1st Cousin, 2nd C. = 2nd Cousin, 3rd C. = 3rd Cousin) and for individuals from populations with small effective population size.

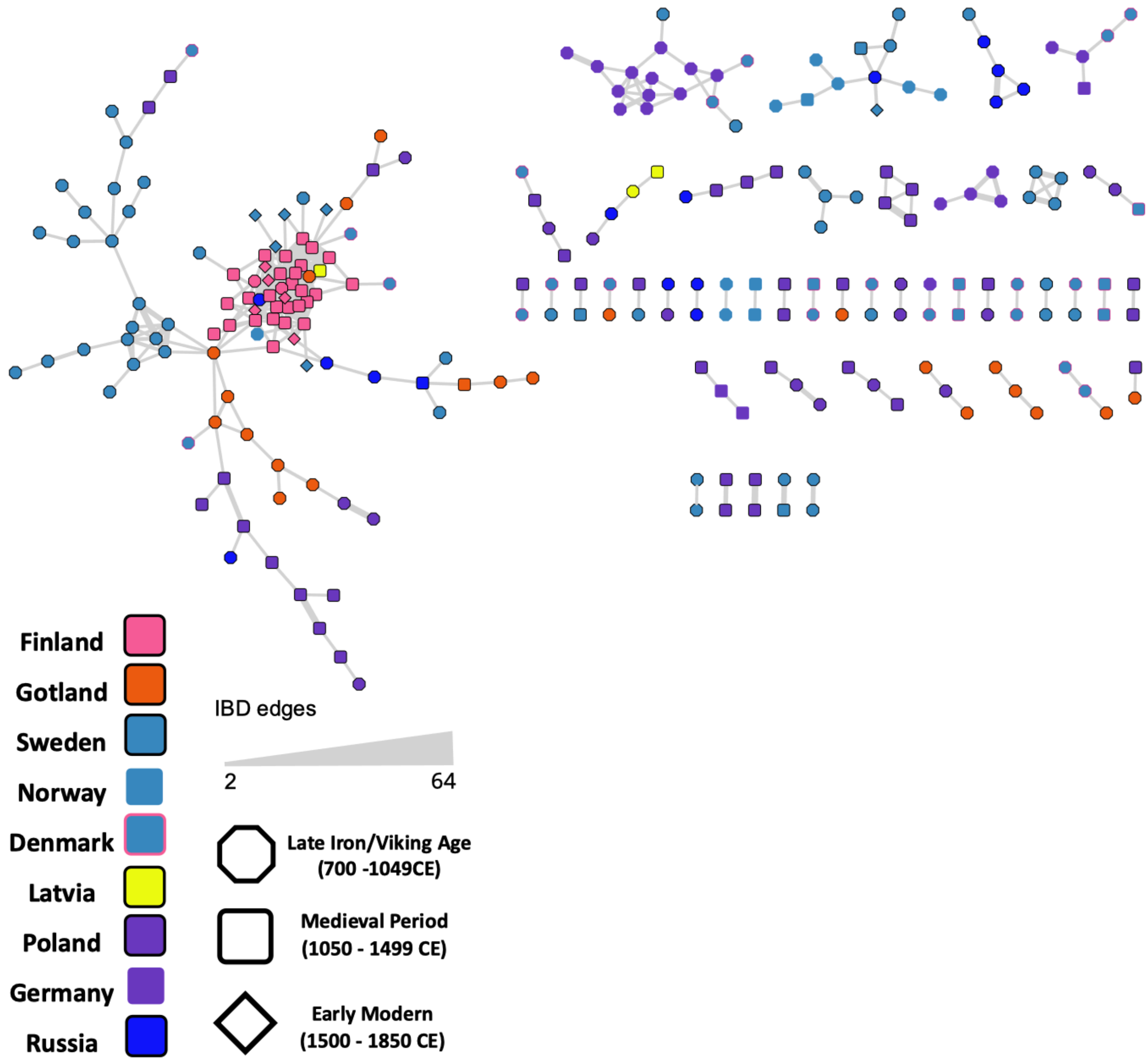

Fig. S8: A contiguous circum-Baltic Identity-By-Descent (IBD) network and multiple satellite networks showing inter-regional connectivity between ancient individuals ranging from the Viking Age to the Early Modern Period. Segments of shared IBD ≥ 8 cM (centiMorgans) called using ancIBD and visualised using Cytoscape. Edge widths (grey lines) are scaled by the total number of IBD segments shared by a pair of individuals, with a thicker edge indicating that the pair share a greater number of IBD segments than those pairs with thinner connecting edges. If two individuals are not connected, then they do not share IBD segments. A prefuse force directed layout is applied to the entire network graph (contiguous and satellite networks) which causes individuals that share more IBD segments to be pushed more closely together.

Individuals colour-coded by their region of burial and age indicated by shape of node. Satellite networks represent pairs, trios, or larger groups of individuals that are connected by shared IBD segments, but are not themselves connected to the larger contiguous network. Satellite networks manually arranged to improve clarity of the main network.

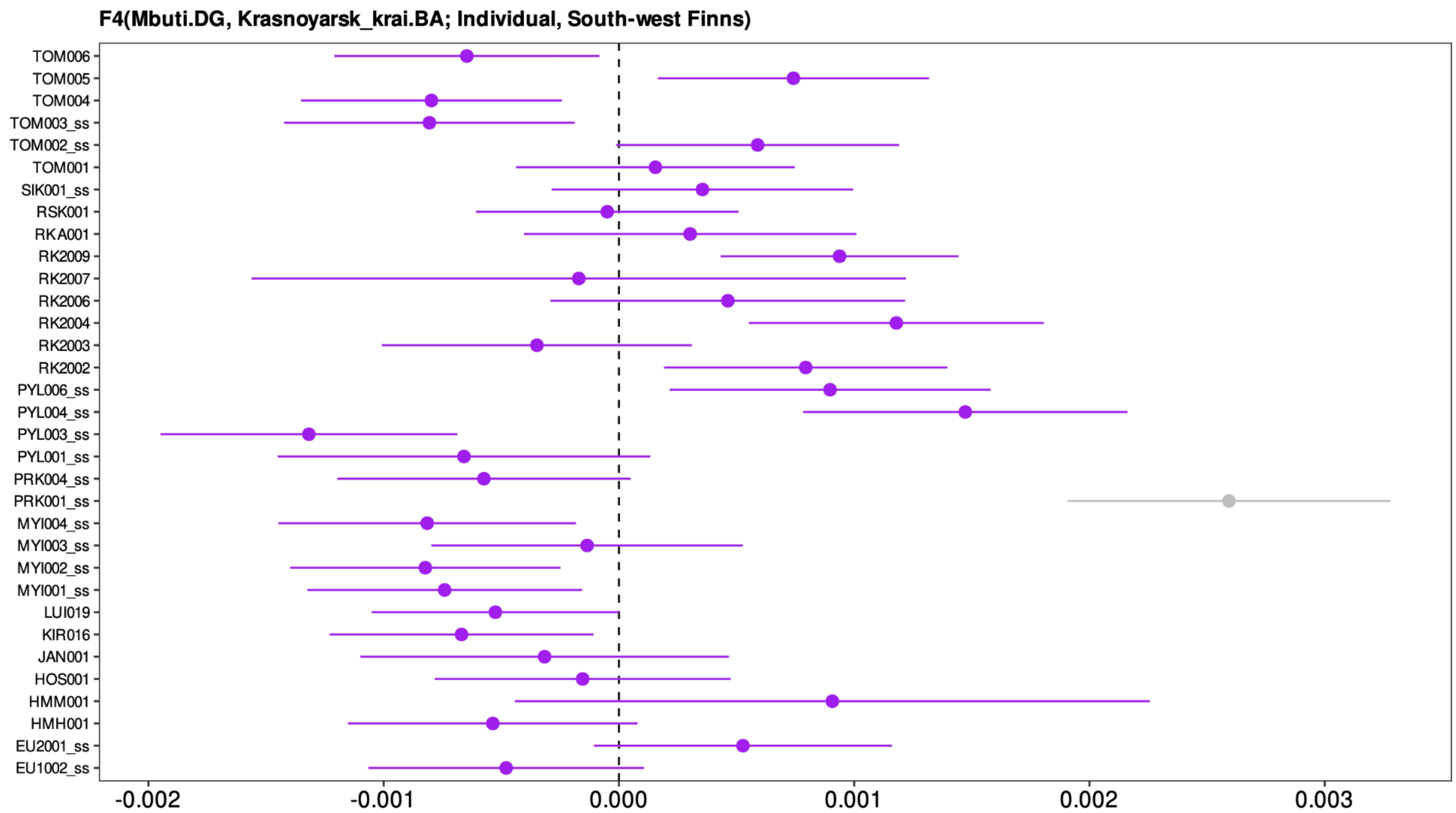

Fig. S9: Individual-level f_4-statistics for assessing excess allele sharing between ancient individuals from southwestern Finland and an ancient source of Siberian ancestry from Bronze Age Siberia (Krasnoyarsk_krai.BA), relative to present-day southwestern Finns. Increasingly negative values indicate greater affinity (i.e., allele sharing) with the ancient source relative to present-day southwestern Finns, and vice-versa. Purple indicates that an individual did not display significantly different levels of allele sharing with the ancient source compared to the present-day ($|Z|<3$). See Additional File 1: Table S17.

Fig. S10: ADMIXTURE plot of mean ancestry components for 92 populations or individuals. k ranged from 2-12, best k ranged from 7 to 9. See Additional File 1: Table S18. See Additional File 4 for high-resolution figure.

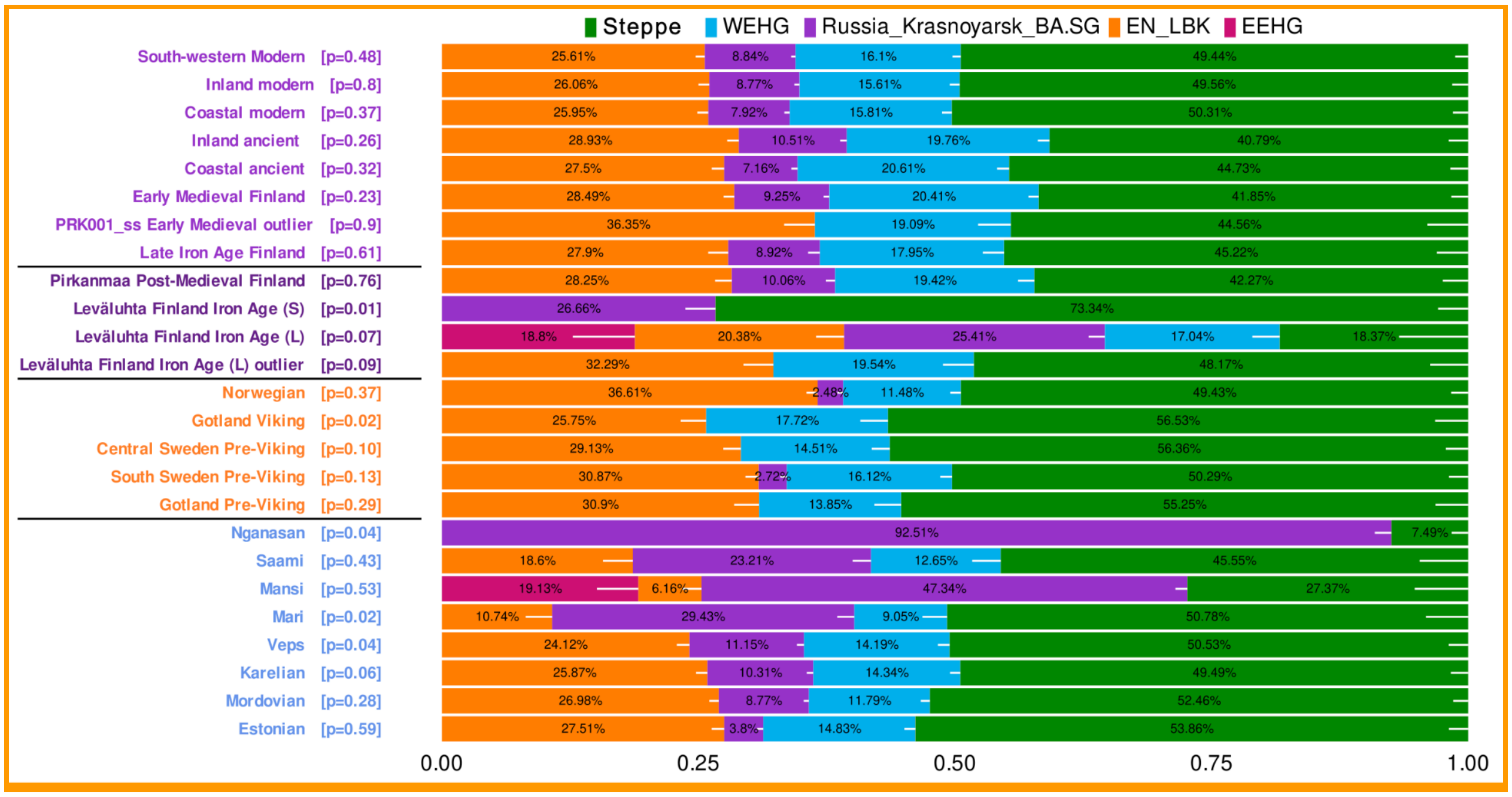

Fig. S11: Distal ancestry modelling using qpAdm. We compare analysis clusters consisting of individuals from this study against all previously published ancient individuals from Finland, present-day southwestern Finns, Sámi in Finland, other extant Finno-Ugric populations, and geographically-proximate or historically-relevant Scandinavian present-day and ancient populations. Reading top to bottom within each geographic subset, populations are ordered from the youngest to oldest. Right populations were Ethiopia_4500BP.DG, CHG (Caucasus Hunter-Gatherer), Russia_CentralYakutia_LN.SG, ANE (Ancient North Eurasian), Villabruna_HG, Anatolian Neolithic Farmer. See Additional File 1: Tables S14, S19.
