# Supplementary material for: Population genomics, demography, and circum-Baltic connectivity of Early Medieval southwestern Finland
Source: Genome Biol. 2026 Sep 15;27:288. doi: 10.1186/s13059-026-04277-7 (PMC13576218; doi:10.1186/s13059-026-04277-7)
Supplement: Supplementary file 2 [file 13059_2026_4277_MOESM3_ESM.pdf]

Text S1: Archaeological details of studied individuals

compiled by Dr Ulla Nordfors

(1) Site: Eura Vanha Kansakoulu (Kaunismäki) 50010016
Abbreviation: EU1, EU2
Description: In 1987, 24 inhumation graves were excavated. Finds were recovered from 8 graves, with the majority concentrated in two of them. The material included spearheads, textile remains, rings, ceramic sherds, and human and animal bones. Further excavations in 1991–92 revealed 7 additional graves. These were oriented from west-southwest to east-northeast and included one double burial. Finds included coins, spearheads, and brooches. The graves date to the late Viking Age and the Crusade Period (9th - 12th century CE).
Individual studied: Finnish Heritage Agency number KM 24007:86
Laboratory ID: EU1002_ss
Details: Grave 2/1987. Grave goods included two Viking-Age spearheads and a bronze finger-ring. There was an animal, possibly a dog, buried at the foot end. The find catalogue mentions the skeletal material found in the excavation has been treated with bone glue.

Individual studied: Finnish Heritage Agency number KM 27176:38
Laboratory ID: EU2001_ss
Details: Grave 1/1992. First of the individuals buried in a double grave. The individuals had been buried in a coffin, with finger rings on their hands, and dressed in clothes decorated with bronze spirals. Two spearheads and a sickle were found in the coffin.

Individual studied: Finnish Heritage Agency number KM 27176:37
Laboratory ID: EU2002_ss
Details: Grave 1/1992. Second of the individuals buried in a double grave. The individuals had been buried in a coffin, with finger rings on their hands, and dressed in clothes decorated with bronze spirals. Two spearheads and a sickle were found in the coffin.

(2) Site: Eura Käräjämäki-Osmanmäki 50010027
Abbreviation: KR1
Description: The oldest graves at the site are cremation burials dating to the 5th century CE. First inhumation burials appear during the 6th century CE, and were continued until the Viking Age (11th century CE), possibly even until Crusade Period (12th century CE). Bone preservation is extremely poor.

Individual studied: Finnish Heritage Agency number KM2700:55
Laboratory ID: KR1002
Details: A grave excavated in 1890. The grave goods included bronze arm rings, an Arabic silver coin likely worn as a pendant, as well as sheathed knife and bronze spiral ornaments attached to textiles.

(3) Site: Eura Luistari 50010032

Abbreviation: LUI

Description: The largest excavated Late Iron Age/Early Medieval inhumation cemetery in Finland. Continuously used from the 6th century CE to 13th/14th centuries CE. There are ~1300 graves at the site, most unfurnished, bone preservation extremely poor.

Individual studied: Finnish Heritage Agency number KM22346:390

Laboratory ID: LUI019 or TU481_UDGh

Details: Pars petrosa sampled. Grave 480. Grave goods included a knife, bronze finger ring, bronze spiral ornaments, axe, scythe, and a spearhead. Radiocarbon dates suggest the grave dates to the latter part of the Early Medieval Period but based on the grave goods, it is potentially from earlier in the period as well. Dietary isotope analyses indicate that the individual was likely non-local, possibly originating from a more distant area within Finland or an area around the Baltic Sea (Danielisová et al. 2025).

(4) Site: Hämeenlinna Honkaliini 1 401010009

Abbreviation: HMH

Description: The site consists of a Viking Age (~800-1050 CE) cremation cemetery, in which at least one inhumation burial has been dug in the Crusade Period (1050-1250 CE). The site was excavated in 1932.

Individual studied: Finnish Heritage Agency number KM 9727:2

Laboratory ID: HMH001

Details: Tooth 27 sampled (82.3 mg). Grave 1/1932. The unburnt human bones were not found in anatomical order, and their position was not documented. According to Nils Cleve (1933), the inhumation burial had possibly included a ceramic vessel and a bronze double-spiral chain-distributor, which have occasionally been used as pendants (Moilanen 2021: 151).

(5) Site: Hämeenlinna Männistönmäki 83010022

Abbreviation: HMM

Description: The site contains both cremation and inhumation burials. The first human remains were discovered in spring 1933 in a sand pit, followed by more bones and Viking Age artefacts that likely originated from an earlier cremation cemetery. The inhumation graves had been dug through cremation layers, as indicated by sooty soil, cremated bones, and melted bronze and bead fragments. The inhumation graves were never properly excavated and documented. In 1999, pipeline work revealed a sword and at least three disturbed graves, of which one (1/1999) was excavated (Moilanen 2021: 153-4).

Individual studied: Finnish Heritage Agency number KM31692:18

Laboratory ID: HMM001

Details: Grave 1/1999. Fragments of cranium were found from the opposite ends of the grave, but because the bones were poorly preserved, it is impossible to make definitive interpretations on the context. There could have been two individuals in the grave (heads at opposite directions as in Valkeakoski Toppolanmäki), additional bones in the grave (like several graves at Hollola Kirkkailanmäki), or the bones could have been commingled due to secondary disturbance. The

objects recovered from the grave included a knife, round pendant, silver filigree bead, and a carnelian bead originating from a cremation cemetery.

(6) Site: Hämeenlinna Pahnainmäki 999000258
Abbreviation: HMP
Description: A Viking Age (9-11th century CE) cremation cemetery, into which at least two Crusade Period (11th-12th century CE) inhumation burials were later inserted. Both inhumations contained artefacts showing signs of exposure to fire (Moilanen 2021: 73, 157-8)
Individual studied: Finnish Heritage Agency number KM6388:34
Laboratory ID: HMP001_ss
Details: Grave 2/1913. Several artefacts found in the grave predate the burial by decades and in some cases by centuries. The Appelgren F–type brooch was already broken and lacked its fastening mechanism, making it unusable as a garment fastener. The chain-distributor represents a Permian type characteristic of the Merovingian and Viking periods. The neck-ring fragments were deliberately arranged around the neck despite having been broken prior to deposition and placed only after the body had been lowered into the grave. These items cannot be interpreted as elements of dress worn in life, but as objects intentionally arranged on the corpse. The knife recovered from the pelvic area may be the only artefact contemporary with the burial itself. The assemblage indicates a conscious re-use of older cremation-cemetery material that was employed to create a symbolic imitation of dress and jewellery (Moilanen 2021: 73, 157-8)

(7) Site: Hattula Ruskeenkärki 82010021
Abbreviation: RSK
Description: Viking Age (9th century CE) burial cairn with at least three individuals (two adults and a child, Moilanen 2021: 118). The bodies have been placed on the ground and covered with large stones. The site is located on a steep, stony slope, somewhat far from arable land and known settlement sites. In previous literature, the site has been interpreted as a temporary burial, a burial of crime victims, or a burial of strangers in the community (Huurre 1976: 31–33; Keskitalo 1963: 36), but it can also represent an ordinary inland burial cairn (c.f. Taavitsainen 2003). The site was excavated in 1955.
Individual studied Finnish Heritage Agency number KM13777:10
Laboratory ID: RSK001
Details: Right pars petrosa of adult sampled (11710 mg). Burial B/1955 in the cairn has been interpreted as a double burial of an adult and a child (Moilanen 2021). The objects associated with the adult individual include bronze spiral ornaments, ~125 beads, a bronze neck-ring, knife, and two round convex brooches.

(8) Site: Hollola Kirkkailanmäki 98010014
Abbreviation: KIR
Description: At least 140 graves have been excavated or documented at the site between 1935-1991. The bones from ~109 graves excavated in 1935 and 1937 were left in the ground and not catalogued. Only bones from 22 graves excavated between 1978-1979 have been catalogued in the National Museum of Finland's collections. The oldest graves at the cemetery date to ~1000 CE

but the site was continuously used until the end of the 14th century CE, possibly until the beginning of the 15th century CE (Moilanen 2021: 51; 121). Therefore, the majority of the graves are Medieval. Due to the dating, the site can be considered a village cemetery from the Catholic period. Most of the inhumation graves are unfurnished but some contain cremation burials that have been placed on the corpses or in their own grave pits in the vicinity of the inhumation graves.

Individual studied: Finnish Heritage Agency number KM21112:30

Laboratory ID: KIR016

Details: Pars petrosa sampled. Grave 15/1979. 1-3 year old infant buried in a coffin with an iron handle. Likely one of the youngest graves in the cemetery. No artefacts.

(9) Site: Hollola Santahaudanmäki 283010003

Abbreviation: HOS

Description: The site has never been properly excavated, but finds from at least three inhumation burials have been recovered from the site. At least two of the graves included sharp objects that had been struck into the coffins or wooden structures in the graves (Moilanen 2021: 149).

Individual studied: Finnish Heritage Agency number KM23043:8

Laboratory ID: HOS001

Details: Left pars petrosa sampled (66 mg). Grave 1/1985. An Early Medieval (~1200 CE) double burial with a coffin or a wooden structure. A bent spearhead and three fragmentary spearheads (and possibly also a fragmentary piece of a sword blade) were struck into these structures. An axe was placed under a skull of one of the individuals. The sampled skull possibly has a perimortem fracture in the left side of the parietal and occipital bone. Birch bark was used as a padding in the coffin. (Moilanen 2021: 149).

(10) Site: Janakkala Kinnari 1 165010026

Abbreviation: HAK

Description: Four partially disturbed graves were excavated at the yard of Kinnari House in Janakkala, just below the Kiianlinna hillfort in 1929-30 (Moilanen 2021: 159).

Individual studied: Finnish Heritage Agency number KM9199:4

Laboratory ID: HAK001

Details: A loose skull belonging to a 10-12-year old child, possibly from a disturbed grave, since part of the mandible has been coloured green by a bronze artefact

(11) Site: Janakkala Makasiininmäki 165010047

Abbreviation: JAN

Description: Finds from both ~4-6 centuries CE and Early Medieval (~11th-13th centuries CE) inhumation burials have been found from the site, mostly in construction works and therefore, the graves have not been documented properly. The site also includes a cremation cemetery from ~600-1050 CE. (Moilanen 2021: 163-164).

Individual studied: Finnish Heritage Agency number KM11938:3

Laboratory ID: JAN001

Details: Left pars petrosa sampled (74.3 mg). Grave 1/1948. A triple burial of two adults and a child (Moilanen 2021). The grave included at least one oval convex brooch. Dates to ~1156-1231 CE. The sampled bone belongs to one of the adults.

(12) Site: Nokia Hakamäki 536010021
Abbreviation: PRK
Description: First grave finds were discovered in 1921 during house foundation work. Later, more finds were collected from six separate locations, possibly representing at least six individual graves. In 1922, Aarne Europaeus excavated three additional graves under challenging conditions that made documentation difficult: the work was carried out within the house foundation filled with wood chips, and the soil was frozen above the burial layer. Torrential rain turned the site to mud, and Europaeus also noted his own fatigue and distraction due to poor living conditions in his lodgings (Moilanen 2021: 175).

Individual studied Finnish Heritage Agency number KM7955:13
Laboratory ID: PRK001_ss
Details: Finds from approximately 5 different inhumation graves found in 1921. Detailed contextual information is not available.

Individual studied Finnish Heritage Agency number KM8073:8
Laboratory ID: PRK003_ss
Details: Grave 2/1922. A spearhead with a silver-inlaid socket was encountered first during excavation, directly above the burial. It is bent and preserves traces of wood, indicating that it had been deliberately struck through the lid. Grave-goods possibly include a whetstone.

Individual studied Finnish Heritage Agency number KM8073:14
Laboratory ID: PRK004_ss
Details: Grave 3/1922. The body was covered with birch bark and two layers of stones. Penannular brooches were found on the shoulders, one of them a distinct Baltic type with a cross motif (Moilanen 2021: 177).

(13) Site: Raisio Kansakoulunmäki 680010001
Abbreviation: RKA or RK2
Description: The cemetery dates to ~12th-13th centuries CE and it has been considered a Christian village cemetery based on the large number of unfurnished burials. ~50 graves have been excavated from the site.

Individual studied (1 of 7): Finnish Heritage Agency number KM14676:128
Laboratory ID: RK2002
Details: Right pars petrosa sampled (73.3 mg). Grave 3/1959 included several bronze spiral ornaments from clothes, apron, and temple decorations. The deceased had a bronze finger ring, a necklace of over 14 glass and bone beads, and a sheathed knife.

Individual studied (2 of 7): Finnish Heritage Agency number KM14676:264
Laboratory ID: RK2003
Details: Left pars petrosa sampled (56 mg). Grave 11/1959 included a knife in a leather sheath and the remains of a coffin.

Individual studied (3 of 7): Finnish Heritage Agency number KM14676:196
Laboratory ID: RK2004
Details: Right pars petrosa sampled (60.5 mg). Grave 6/1959. Unfurnished burial in a coffin.

Individual studied (4 of 7): Finnish Heritage Agency number KM14676:290
Laboratory ID: RK2006
Details: Left pars petrosa sampled (58.6 mg). Grave 16/1959. Potentially a disturbed context.

Individual studied (5 of 7): Finnish Heritage Agency number KM14676:44a
Laboratory ID: RK2007
Details: Right pars petrosa sampled (60 mg). Grave 2/1959 included bronze spiral ornaments from clothes, apron, and temple spiral ornaments, three penannular brooches of which two have been used on the shoulders as dress brooches. The burial had been made in a coffin.

Individual studied (6 of 7): Finnish Heritage Agency number KM14676:302
Laboratory ID: RK2009
Details: Left pars petrosa sampled (60.3 mg). Grave 17/1959. Burial in a coffin. The grave included bronze spiral ornaments from apron and temple jewellery.

Individual studied (7 of 7): Finnish Heritage Agency number KM14275:157
Laboratory ID: RKA001
Details: Right pars petrosa sampled (59.9 mg). Bones from a disturbed grave in area A1 excavated in 1957.

(14) Site: Salo Halikko Rikalanmäki 73010022
Abbreviation: SIK
Description: Crusade Period (11th - 13th century CE) inhumation cemetery with ~50 graves. Among the finds were numerous Crusade Period swords, jewellery, utensils and tools, ceramics, textile fragments, and bronze spiral ornaments used as dress decorations. Adjacent to the inhumation cemetery is an older cremation cemetery. The site was excavated in the 1950s.
Individual studied Finnish Heritage Agency number KM12690:163a
Laboratory ID: SIK001_ss
Details: Grave Ga. Bones from cranium and teeth, not in an anatomical order. Likely from a disturbed burial.

(15) Site: Salo Perniö Yliskylän hautausmaa 586010010
Abbreviation: PYL

Description: During the expansion of the Yliskylä chapel cemetery in the summer of 1893, a cremation burial dating to the Early Iron Age was discovered. In 1895, eleven Early Medieval (12th - 13th century CE) inhumation graves were examined at the site. The graves were dug in sandy soil and arranged in a north–south orientation.
Individual studied Finnish Heritage Agency number KM3106:32
Laboratory ID: PYL001_ss
Details: Grave 1/1895. Grave goods included a knife.

Individual studied Finnish Heritage Agency number KM2912:114
Laboratory ID: PYL003_ss
Details: Grave 8/1893. Grave goods included a penannular brooch and glass beads.

Individual studied Finnish Heritage Agency number KM2912:117
Laboratory ID: PYL004_ss
Details: Grave 9/1893. Grave goods included a ring brooch of Baltic type and bronze spiral ornaments from the clothes of the deceased.

Individual studied Finnish Heritage Agency number KM2912:64
Laboratory ID: PYL006_ss
Details: Grave 3/1893. Grave goods included two penannular brooches, an earspoon, armring, bronze spiral decorations attached to textiles, and a necklace of beads and a European coin. The individual was buried in a coffin.

(16) Site: Valkeakoski Toppolanmäki 908010003
Abbreviation: TOM
Description: The site has mainly been excavated in the 1930s, when some of the bones were left *in situ*. Two of the graves initially found in 1936 and 1937 were excavated in 2017 and 2018. At least 15–17 individuals have been buried at the site, all which seem to have been adults. At least two of the graves contained more than one individual (Moilanen 2021: 196).
Individual studied (1 of 6): Finnish Heritage Agency number KM41248
Laboratory ID: TOM001
Details: Tooth 47 sampled (67.8 mg). Grave 3/1937. Initially excavated in 1937, reopened in 2017. Large stones were placed on the corpse that was buried without a coffin. There were no artefacts in the grave, but the grave pit had been padded with mosses (Moilanen et al. 2022).

Individual studied (2 of 6): Finnish Heritage Agency number KM10461:12

Laboratory ID: TOM002

Details: Tooth FDI 48 sampled. Human remains from disturbed graves, discovered during sand extraction prior to the first archaeological investigations in 1936.

Individual studied (4 of 6): Finnish Heritage Agency number KM10461:13

Laboratory ID: TOM003

Details: Pars petrosa sampled. Human remains from disturbed graves, discovered during sand extraction prior to the first archaeological investigations in 1936.

Individual studied (4 of 6): Finnish Heritage Agency number KM41564:2

Laboratory ID: TOM004

Details: Tooth 44 sampled (74 mg). Grave 2/1936. Initially excavated in 1936, reopened in 2018. A multiple burial of three individuals in the same coffin. There were no artefacts in the grave, but according to documentation done in 1936, a broken iron object had been struck through the coffin lid.

Individual studied (5 of 6): Finnish Heritage Agency number KM41564:3

Laboratory ID: TOM005

Details: Tooth 46 sampled (71 mg). Grave 2/1936. Initially excavated in 1936, reopened in 2018. A multiple burial of three individuals in the same coffin. There were no artefacts in the grave, , but according to documentation done in 1936, a broken iron object had been struck through the coffin lid.

Individual studied (6 of 6): Finnish Heritage Agency number KM41564:4

Laboratory ID: TOM006

Details: Tooth 38 sampled (66.1 mg). Grave 2/1936. Initially excavated in 1936, reopened in 2018. A multiple burial of three individuals in the same coffin. There were no artefacts in the grave, , but according to documentation done in 1936, a broken iron object had been struck through the coffin lid.

(17) Site: Ylöjärvi Mikkola 980010024
Abbreviation: MYI
Description: Approximately 18 Early Medieval (ca. 11th-13th centuries CE) burials are known from the site, most of which were excavated in 1959 and during the 1970s. The cemetery has been partially destroyed by later land use, and some features have later been interpreted as not graves but remains of historical-period activity, including a few animal burials. Burnt artefacts were deliberately placed in the grave fills, likely originating from an older cremation cemetery located about 50 meters north of the inhumation graves. (Moilanen 2021: 202).

Individual studied: Finnish Heritage Agency number KM19901:227
Laboratory ID: MYI001_ss
Details: Grave 2b/1976. Multiple burial consisting of a double grave and a third grave separated by arrowheads that had been struck in the earth in a vertical position. This individual was buried in the double grave. Grave goods included two firesteels on the left side of the pelvis, two knives, and flint.

Individual studied: Finnish Heritage Agency number KM19901:238
Laboratory ID: MYI002_ss

Details: Grave 3/1976. Multiple burial consisting of a double grave and a third grave separated by arrowheads that had been struck in the earth in a vertical position. This individual was buried in the third grave. Grave goods included three arrowheads on the right shoulder, axe and firesteel on the right side of the pelvis, knife and flint between the legs, by the left femur.

Individual studied: Finnish Heritage Agency number KM19901:208
Laboratory ID: MYI003_ss
Details: Grave 1/1976. A grave of a ca. 50-year old male. The grave included a sword, which had been placed in the grave tip pointing towards the head. Two spearheads were struck through the coffin lid (Moilanen 2021: 204).

Individual studied: Finnish Heritage Agency number KM19901:217
Laboratory ID: MYI004_ss
Details: Grave 2a/1976. Multiple burial consisting of a double grave and a third grave separated by arrowheads that had been struck in the earth in a vertical position. This individual was buried in the double grave. Grave goods included three arrowheads with transverse points on the left shoulder. Axe on the left side of the pelvis, knife between the legs, by the right femur. Ring on the left hand.
