# Supplementary material for: Population genomics, demography, and circum-Baltic connectivity of Early Medieval southwestern Finland
Source: Genome Biol. 2026 Sep 15;27:288. doi: 10.1186/s13059-026-04277-7 (PMC13576218; doi:10.1186/s13059-026-04277-7)
Supplement: Supplementary file 3 [file 13059_2026_4277_MOESM4_ESM.pdf]

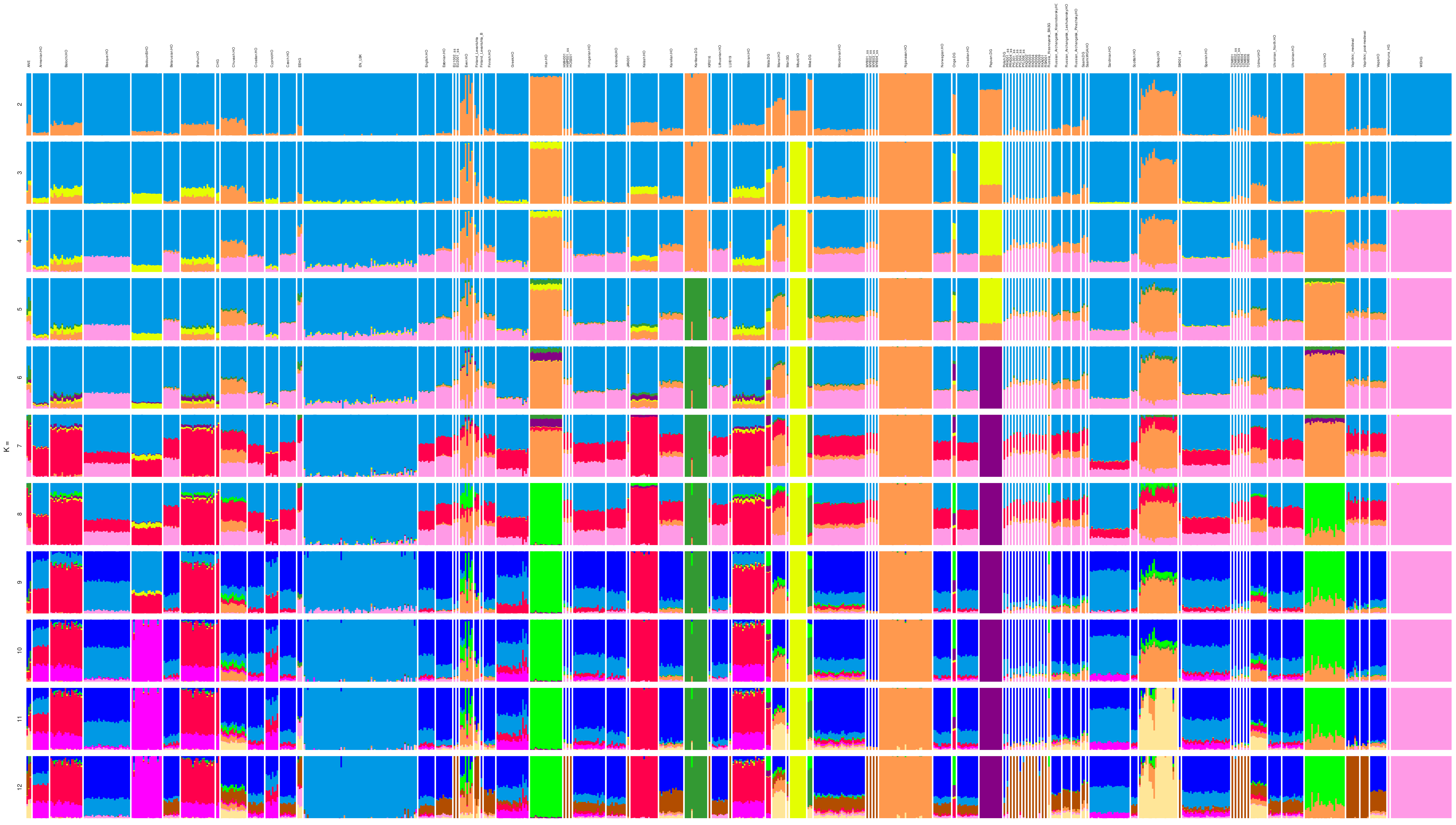

ANE
Armenian.HO
Balochi.HO
Basque.HO
BedouinB.HO
Belarusian.HO
Brahui.HO
CHG
Chuvash.HO
Croatian.HO
Cypriot.HO
Czech.HO
EEHG
EN_LBK
English.HO
Estonian.HO
EU1002_ss
EU2001_ss
Even.HO
Finland_Levanluhta
Finland_Levanluhta_B
Finnish.HO
Greek.HO
Han.HO
HMH001
HMP001_ss
HOS001
Hungarian.HO
Icelandic.HO
JAN001
Kalash.HO
Karelian.HO
Karitiana.DG
KIR016
Lithuanian.HO
LUI019
Makrani.HO
Mala.DG
Mansi.HO
Mari.SG
Mbuti.HO
Mixe.DG
Mordovian.HO
MYI001_ss
MYI002_ss
MYI003_ss
MYI004_ss
Nganasan.HO
Norwegian.HO
Onge.DG
Orcadian.HO
Papuan.DG
Polish.DG
PRK001_ss
PRK004_ss
PYL001_ss
PYL003_ss
PYL004_ss
PYL006_ss
RK2002
RK2003
RK2004
RK2006
RK2009
RKA001
RSK001
Russia_Krasnoyarsk_BA.SG
Russian_Archangelsk_Krasnoborsky.HO
Russian_Archangelsk_Leshukonsky.HO
Russian_Archangelsk_Pinezhsky.HO
Saami.DG
Saami.WGA.HO
Sardinian.HO
Scottish.HO
Selkup.HO
SIK001_ss
Spanish.HO
TOM001
TOM002_ss
TOM003_ss
TOM004
TOM005
TOM006
Udmurt.HO
Ukrainian_North.HO
Ukrainian.HO
Ulchi.HO
Vapriikki_medieval
Vapriikki_post-medieval
Veps.HO
Villabruna_HG
WEHG
K =
2
3
4
5
6
7
8
9
10
11
12
